# Biomolecular mechanisms of iron oxidation in prokaryotes: a protein focused review

**DOI:** 10.1093/femsre/fuag055

**Published:** 2026-11-03

**Authors:** Gianmarco D’Antonio, Agnese Roscioni, Alice Romagnoli, Alessandro Becci, Francesca Beolchini, Giovanni Di Muccio, Alessia Amato, Anna La Teana

**Affiliations:** Department of Life and Environmental Sciences, Polytechnic University of Marche, Via Brecce Bianche, 60131 Ancona, Italy; Department of Life and Environmental Sciences, Polytechnic University of Marche, Via Brecce Bianche, 60131 Ancona, Italy; Department of Life and Environmental Sciences, Polytechnic University of Marche, Via Brecce Bianche, 60131 Ancona, Italy; New York-Marche Structural Biology Center (NY-MaSBiC), Department of Life and Environmental Sciences, Polytechnic University of Marche, Via Brecce Bianche, 60131 Ancona, Italy; Department of Life and Environmental Sciences, Polytechnic University of Marche, Via Brecce Bianche, 60131 Ancona, Italy; Department of Life and Environmental Sciences, Polytechnic University of Marche, Via Brecce Bianche, 60131 Ancona, Italy; Department of Life and Environmental Sciences, Polytechnic University of Marche, Via Brecce Bianche, 60131 Ancona, Italy; New York-Marche Structural Biology Center (NY-MaSBiC), Department of Life and Environmental Sciences, Polytechnic University of Marche, Via Brecce Bianche, 60131 Ancona, Italy; Department of Life and Environmental Sciences, Polytechnic University of Marche, Via Brecce Bianche, 60131 Ancona, Italy; Department of Life and Environmental Sciences, Polytechnic University of Marche, Via Brecce Bianche, 60131 Ancona, Italy; New York-Marche Structural Biology Center (NY-MaSBiC), Department of Life and Environmental Sciences, Polytechnic University of Marche, Via Brecce Bianche, 60131 Ancona, Italy

**Keywords:** ferrous iron oxidation, iron-oxidizing prokaryotes, electron transfer proteins, c-type cytochromes, structural bioinformatics, biotechnological applications

## Abstract

Iron oxidation by prokaryotes is a fundamental biogeochemical process with broad environmental and biotechnological significance. Despite growing knowledge of iron oxidation pathways at the systems level, the structural and biochemical characterization of individual proteins directly responsible for ferrous iron [Fe(II)] oxidation remains limited. This review provides a critical survey of the molecular knowledge of proteins directly involved in Fe(II) oxidation across phylogenetically diverse microorganisms (including acidophilic bacteria and archaea and neutrophilic bacteria) spanning aerobic, anaerobic, and phototrophic metabolic strategies. For each protein (Cyc2, Cyt572, Cyc2PV-1, sulfocyanin, FoxC, PioAB complex, MtoAB complex, and Iro), we discuss structural features, cofactor binding and localization, and redox properties, integrating experimental biochemistry with structural predictions. We further provide an overview of how broader computational approaches, including molecular dynamics simulations and quantum methods, also for redox potential calculations, can complement experimental data to yield mechanistic insights into iron oxidation. Finally, we highlight existing knowledge gaps and outline biotechnological applications (including bioleaching, wastewater treatment, bioremediation, and bioelectrocatalysis) that stand to benefit from a deeper molecular understanding of these proteins.

## Teaser

This review synthesizes current structural and biochemical understanding of ferrous iron-oxidizing proteins in prokaryotes, spanning acidophilic to neutrophilic organisms, and discusses their relevance for biotechnological applications, including bioleaching, bioremediation, and bioelectrocatalysis.

## Introduction

Iron represents the most abundant transition metal within the lithosphere and, on a mass basis, ranks among the most prevalent elements on Earth (Johnson et al. 2012). Moreover, it is also ubiquitously found across aquatic environments, reflecting its extensive distribution and underscoring its environmental significance (Huang et al. 2021).

Given its widespread occurrence, iron plays a central role in the global biogeochemical cycling of both major and trace elements, including carbon, oxygen, nitrogen, and sulfur. For this reason, it exerts a wide range of direct and indirect influences on fundamental environmental processes, such as corrosion phenomena, degradation of organic and inorganic compounds, metal mobility, mineral dissolution, nutrient bioavailability, as well as rock weathering and diagenetic transformations, in close interaction with microbial activity (Huang et al. 2021).

Beyond its environmental significance, iron is very important for human society, as its industrial processing accounts for ~95% of global metal production. Furthermore, microbially mediated ferrous iron oxidation has recently gained increasing attention for its exploitation in biotechnological applications, such as in bioleaching processes for metal recovery and in wastewater treatment (Becci et al. 2020, Lee et al. 2022).

In natural environments, iron exists in two main redox states: ferrous iron [Fe (II)] and ferric iron [Fe (III)]. However, its bioavailability is largely controlled by the pH conditions (Kappler et al. 2021). In extremely acidic environments, whether natural or anthropogenically influenced, the oxidative dissolution of sulfide minerals [e.g. pyrite (FeS₂)], represents a major source of dissolved iron. Moreover, under low pH conditions, both ionic forms of uncomplexed iron, and particularly ferric iron, exhibit substantially higher solubility compared to circumneutral pH (Johnson et al. 2012).

Although iron has only two naturally occurring redox states, its environmental speciation, mobility, and reactivity are controlled by a complex interplay of biotic and abiotic processes. Abiotic Fe(II) oxidation proceeds via both homogeneous and heterogeneous pathways, producing reactive oxygen species such as superoxide, hydrogen peroxide and hydroxyl radicals (Barnes et al. 2009). In parallel, microbial activity significantly contributes to iron redox cycling, with Fe(III)-reducing bacteria and various Fe(II)-oxidizing microorganisms catalyzing the transformation of iron under acidic and circumneutral conditions (Lee et al. 2022). This broad distribution of pH conditions, temperatures, and oxygen availability under which biotic iron oxidation occurs emphasizes the evolutionary adaptability of iron-oxidizing microorganisms, which have successfully colonized diverse niches by coupling iron redox transformations to distinct metabolic and environmental strategies (Lee et al. 2022).

An additional key factor influencing iron chemistry, and consequently its exploitation by prokaryotes as an energy source, is the redox potential of the Fe(II)/Fe(III) couple. This parameter is primarily governed by environmental pH, which strongly controls ferric iron solubility and, to a minor extent, that of ferrous iron, as well as by the presence or absence of ligands capable of complexing iron species. The Fe(II)/Fe(III) redox couple exhibits its highest positive potential of ~+0.77 V under extremely acidic conditions, where both oxidation states remain soluble. In contrast, at circumneutral pH, iron typically occurs in poorly soluble mineral forms, such as ferrous carbonates and ferric hydroxides, which are associated with markedly lower redox potentials (Hedrich et al. 2011).

Despite its limited solubility and reduced bioavailability under circumneutral pH conditions, iron has been evolutionarily conserved as an essential cofactor in a wide array of proteins and enzymes due to its unique redox properties, arising from the interconversion between Fe(II) and Fe(III). These chemical features enable it to participate in fundamental metabolic processes, including oxygen handling, redox catalysis, and electron transfer. Consequently, living organisms have developed highly specialized mechanisms for iron acquisition, transport, storage and regulation (e.g. siderophores and ferritin), allowing cells to maintain iron in a soluble, bioavailable, and non-toxic form (Ilbert and Bonnefoy 2013).

To date, at least eight distinct microbial pathways or models for Fe(II) oxidation have been described, and ongoing comparative genomic and functional studies continue to identify novel genes and gene clusters associated with this metabolism across an expanding range of taxa.

However, in contrast to the growing understanding at the pathway level, the detailed biochemical and structural characterization of individual proteins directly involved in iron oxidation remains relatively limited (Ilbert and Bonnefoy 2013, Li L et al. 2023). This gap largely reflects the experimental challenges associated with the isolation, purification, and functional analysis of iron-oxidizing proteins, which are frequently membrane-associated and therefore difficult to study using conventional biochemical techniques.

In recent years, iron-oxidizing microorganisms have been increasingly investigated and applied in environmental and ecological contexts, often without requiring a detailed molecular understanding of the mechanisms underlying iron oxidation (Schmidt et al. 2010, Fleming et al. 2014, Becci et al. 2020). Therefore, a deeper molecular understanding of these proteins is not only required to resolve fundamental aspects of microbial iron metabolism, but also to support the development of more targeted and efficient biotechnological and environmental applications based on iron-oxidizing enzymes.

In this context, the aim of this review is to provide a critical overview of the current molecular knowledge of proteins directly involved in ferrous iron oxidation, highlighting existing gaps in the literature, discussing potential strategies to address these knowledge gaps and outlining prospective biotechnological applications that could harness these proteins.

## Overview of the main iron oxidizing microorganisms

According to Hedrich et al. (2011), iron (II)-oxidizing microorganisms can be broadly classified into four major physiological groups: aerobic acidophiles, aerobic neutrophiles, anaerobic neutrophilic phototrophs, and anaerobic neutrophiles that couple Fe (II) oxidation to the reduction of nitrate, perchlorate, or chlorate.

Among the diverse Fe(II) oxidizers described to date, only a limited number of microorganisms have been investigated in sufficient molecular detail to allow the identification of proteins involved in, or associated with, Fe(II) oxidation (Li L et al. 2023). These taxa, for which molecular information is currently available, are briefly introduced in the following section. Moreover, at the end of the chapter, we report in Table 1 a summary of the main organism, with the proposed iron oxidation protein and level of evidence supporting their role in Fe(II) oxidation. The microbiological terms used are defined in the Glossary (Supplementary Material).

**Table 1:** Physiological features of the microorganisms in which Fe(II) oxidation pathways are discussed in this review.

Microorganism	Domain	pH	T	Carbon & Energy source	Fe(II) electron acceptor	Proposed iron oxidation protein	Evidence for iron oxidation
*Acidithiobacillus ferroxidans*	E	A	M	Inorganic	O_2_	Cyc2	Experimentally validated: purified protein; Fe(II)-dependent reduction; spectroscopic characterization; mutagenesis (Yarzábal A et al. 2002, Castelle C et al. 2008, Su et al. 2025)
*Acidithiobacillus ferridurans*	E	A	M	Inorganic	O_2_	IRO	Experimentally validated: heterologous expression; Fe(II)-dependent electron transfer; kinetic analysis (Liu Y et al. 2023)
*Leptospirillum ferriphilum*	E	A	M	Inorganic	O_2_	Cyt572	Experimentally validated: purified protein; spectroscopic and redox characterization (Jeans et al. 2008)
*Ferroplasma acidiphilum*	AR	A	M	Inorganic/organic	O_2_	Sulfocyanin	Experimentally supported at the complex level: Fe(II)-oxidizing activity and O₂ consumption in sulfocyanin-containing complexes. (Castelle CJ et al. 2015)
*Metallosphaera sedula*	AR	A	T	Inorganic/organic	O_2_	FoxC	Inferred from indirect evidence: proteomic identification; Fe(II)-dependent transcriptional upregulation; comparative genomics; structural homology (Counts et al. 2022, Kozubal et al. 2011)
*Sideroxydans lithotrophicus*	E	N	M	Inorganic	O_2_	MtoA	Experimentally validated: heterologous expression; Fe(II)-dependent reduction; ligand-dependent Fe(II) oxidation activity (Liu J et al. 2012)
*Mariprofundus ferrooxydans*	E	N	M	Inorganic	O_2_	Cyc2PV-1	Experimentally validated: proteomic identification; heterologous expression; Fe(II)-dependent reduction; spectroscopic characterization (Barco et al. 2015, Keffer et al. 2021)
*Rhodopseudomonas palustris TIE-1*	E	N	M	Inorganic/organic	RC	PioA	Experimentally validated: gene deletion; loss of Fe(II)-oxidizing activity; biochemical characterization; Fe(II)-dependent reduction (Jiao and Newman 2007, Gupta et al. 2019)

Legend: E, Eubacteria; AR, archaea; A, acidophilic; N, neutrophilic; M, mesophile; and T, thermophile. More details on the proteins can be found in Supplementary Table S1, the Glossary for the terms is reported in Supplementary Materials.

### Aerobic acidophilic Fe(II) oxidizers

Aerobic acidophilic Fe(II) oxidizers represent the most widespread group and exhibit remarkable phylogenetic diversity. They are found across multiple bacterial lineages, including Gram-negative taxa such as *Nitrospira, Acidobacteria, Betaproteobacteria*, and *Gammaproteobacteria*, as well as Gram-positive *Firmicutes* and *Actinobacteria*.

*Acidithiobacillus ferrooxidans*, e.g. is a Gram-negative, acidophilic, aerobic chemoautotroph. The organism relies exclusively on CO₂ as its carbon source and derives metabolic energy from the oxidation of ferrous iron, elemental sulfur, and other reduced sulfur compounds (Valdés et al. 2008). Regarding Fe²⁺ oxidation, the majority of electrons are transferred to molecular oxygen following the redox potential gradient, whereas a smaller fraction flows against the gradient to generate NADH. These two routes are referred to as the downhill and uphill pathways, respectively, and are tightly interconnected (Zhang S et al. 2018).

Other well-characterized acidophiles belong to the genus *Leptospirillum*. Members of the genus are vibrio- or spiral-shaped microorganisms that derive energy from Fe(II) oxidation and fix carbon dioxide. They typically thrive under highly acidic conditions (pH 1.0–2.0) and display optimal growth at temperatures ranging from 30°C to 37°C, although several strains exhibit moderate thermophily. Among them, *L. ferriphilum* is a key component of bioleaching consortia, where it contributes to the solubilization of metals from sulfide ores (Vardanyan et al. 2023).

In addition, several acidophilic Fe(II)-oxidizers have been identified within the archaeal domains *Crenarchaeota* and *Euryarchaeota* (Ilbert and Bonnefoy 2013). Within the *Euryarchaeota*, the genus *Ferroplasma*, exhibits metabolic features closely related to those observed in other archaeal members of the order *Sulfolobales* and in bacterial iron oxidizers of the genera *Acidithiobacillus* and *Leptospirillum*. This is consistent with the fact that these microorganisms commonly inhabit similar acidic and metal-rich ecological niches (Castelle CJ et al. 2015). For example, *Ferroplasma acidiphilum* is extremely acidophilic, growing optimally at pH 1.7, and mesothermophilic. It oxidizes ferrous iron (FeSO₄) and pyrite (FeS₂) and fixes inorganic carbon (Golyshina and Timmis 2005). Among *Crenarchaeota*, the genus *Metallosphaera*, which belongs to the order *Sulfolobales* like *Ferroplasma*, comprises thermoacidophilic archaea capable of aerobic Fe(II) and sulfur oxidation, like *Metallosphaera sedula*. They grow over a broad temperature range (50°C–80°C) and tolerate acidic conditions from pH 1.0 to 6.5. Members of this genus are obligately aerobic and facultatively chemolithoautotrophic. They can solubilize metal ions from various sulfide minerals, including pyrite, chalcopyrite, and sphalerite. In addition, they oxidize elemental sulfur to sulfate, although they are unable to use elemental sulfur as a terminal electron acceptor (Liu LJ et al. 2021).

### Aerobic neutrophilic Fe(II) oxidizers

In contrast to the widespread occurrence of aerobic acidophilic Fe(II) oxidizers, all currently known aerobic neutrophilic Fe(II) oxidizers are restricted to the phylum Proteobacteria (Lee et al. 2022). Freshwater neutrophilic iron oxidizers are predominantly affiliated with the *Betaproteobacteria* such as *Sideroxydans lithotrophicus* (Lee et al. 2022), whereas their marine counterparts belong to a distinct phylogenetic lineage within the class Zetaproteobacteria, represented by *Mariprofundus ferrooxydans* (Emerson et al. 2010). The latter organism promotes iron biomineralization through the production of extracellular twisted stalk-like structures. It represents one of the few marine microorganisms demonstrated to grow exclusively by chemolithoautotrophic metabolism based on Fe(II) oxidation at circumneutral pH, where biological oxidation directly competes with rapid abiotic iron oxidation processes (Barco et al. 2015).

Similarly, *Sideroxydans lithotrophicus* ES-1 is a Gram-negative, neutrophilic Fe(II)-oxidizing bacterium that grows at oxic–anoxic interfaces using Fe(II)-bearing minerals such as siderite (FeCO₃) or FeS as energy sources. Its metabolism is strictly linked to iron oxidation, as it does not utilize organic carbon compounds and does not perform Fe(III) reduction. During growth, Fe(III) oxy- hydr-oxide precipitates form in close association with the cells, although they do not develop the characteristic stalk- or sheath-like structures observed in other Fe(II)-oxidizing bacteria (e.g. *Mariprofundus ferrooxydans*) (Liu J et al. 2012).

### Anaerobic Fe(II) oxidizers

Anaerobic Fe(II) oxidizers display broad phylogenetic heterogeneity. Nitrate-, perchlorate-, or chlorate-dependent Fe(II)-oxidizing microorganisms have been identified among several bacterial classes, including *Alpha*-, *Beta*-, *Gamma*-, and *Deltaproteobacteria*, as well as within the archaeal phylum *Crenarchaeota* (Hedrich et al. 2011).

### Phototrophic anaerobic Fe(II) oxidizers

In contrast, phototrophic anaerobic Fe(II) oxidizers have been detected in members of the *Chlorobia* and in *Alpha*- and *Gammaproteobacteria* but have not yet been reported in Archaea. An example of phototrophic anaerobic iron-oxidizing bacteria (IOB) is *Rhodopseudomonas palustris* TIE-1, which integrates cadmium detoxification with biological nitrogen fixation, thereby facilitating the conversion of atmospheric nitrogen into bioavailable ammonium that can sustain plant growth (Lee et al. 2022). This organism belongs to the group of purple non-sulfur bacteria and exhibits marked metabolic flexibility, as it can shift among photoautotrophic, photoheterotrophic, chemoheterotrophic, and chemoautotrophic metabolic strategies in response to environmental conditions (Li M et al. 2022).

The widespread distribution of Fe(II) oxidation across both Bacteria and Archaea strongly suggests that dissimilatory iron oxidation represents an ancient metabolic strategy. On this basis, it has been hypothesized that anoxic Fe(II)-oxidizing microorganisms may have played a pivotal role in the formation of early Banded Iron Formations during the anoxic stages of Earth’s biosphere (Ilbert and Bonnefoy 2013, Vardanyan et al. 2023).

## Microbial Fe(II) oxidation: diversity of strategies and molecular basis of electron transfer

Li and colleagues report that at least eight iron oxidation pathways have, so far, been described with sufficient molecular resolution and therefore represent model systems illustrating the diverse metabolic strategies employed by iron-oxidizing microorganisms to colonize ecological niches spanning a wide range of environmental conditions (Li L et al. 2023).

Although these Fe(II)-oxidizing pathways exhibit substantial diversity among different microorganisms, they share several overarching organizational features. Iron oxidation typically occurs either in the periplasmic space or at the outer membrane and is characterized by high abundance of redox-active proteins, particularly in Gram-negative bacteria, despite the relatively small redox potential difference between the Fe(II)/Fe(III) and O₂/H₂O couples (Ilbert and Bonnefoy 2013, Li L et al. 2023). In most characterized systems, c-type cytochromes act as the primary Fe(II) oxidases, mediating electron transfer from extracellular Fe(II) to terminal electron acceptors through vertically organized electron transport chains that connect the external environment to the cytoplasmic membrane (He et al. 2017). This strategy confers several physiological advantages, including the maintenance of cytoplasmic pH homeostasis and the prevention of intracellular iron precipitation. Moreover, this spatial organization minimizes intracellular exposure to reactive oxygen species generated through Fe²⁺-mediated Fenton chemistry, thereby protecting cellular macromolecules from oxidative damage (Ilbert and Bonnefoy 2013).

In acidophilic iron-oxidizing microorganisms, the oxidation of ferrous iron supplies electrons that balance proton translocation across the cytoplasmic membrane, necessary for ATP generation. This process prevents intracellular acidification and supports the reduction of molecular oxygen via the downhill electron transfer pathway. In parallel with this energetically favorable route, reducing power in the form of NADH is generated from electrons derived from Fe(II) oxidation. This reaction, however, is thermodynamically unfavorable because the redox potential of the NAD⁺/NADH couple (−0.32 V) is considerably lower than that of the Fe(II)/Fe(III) pair. Consequently, electron transfer from Fe(II) to NAD⁺ must proceed against the electrochemical gradient, constituting the so-called uphill pathway. This reverse electron flow is presumed to be driven by the proton motive force (i.e. electrochemical gradient of protons across a membrane), generated during respiration (Jones and Santini 2023).

### Acidithiobacillus ferrooxidans

*Acidithiobacillus ferrooxidans* represents an exemplary model for elucidating the spatial and functional organization of iron oxidation pathways in acidophilic microorganisms. During Fe(II) oxidation process about 95% of the electrons enter the downhill pathway. Cyc2 is proposed to act as the initial electron acceptor during Fe(II) oxidation given its localization in the outer membrane. Rusticyanin (Rus), a blue copper protein located in the periplasm, together with the c-type cytochrome Cyc1 and a copper-containing protein (Cup), forms an electron transfer complex that conveys electrons from the outer-membrane Cyc2 to the aa₃-type cytochrome oxidase complex (CoxABCD) located in the cytoplasmic membrane. This terminal oxidase subsequently transfers electrons to molecular oxygen, catalysing its reduction to water and coupling this process to proton translocation for ATP synthesis (Fig. 1a) (Zhang S et al. 2018, Li L et al. 2023).

**Figure 1:**
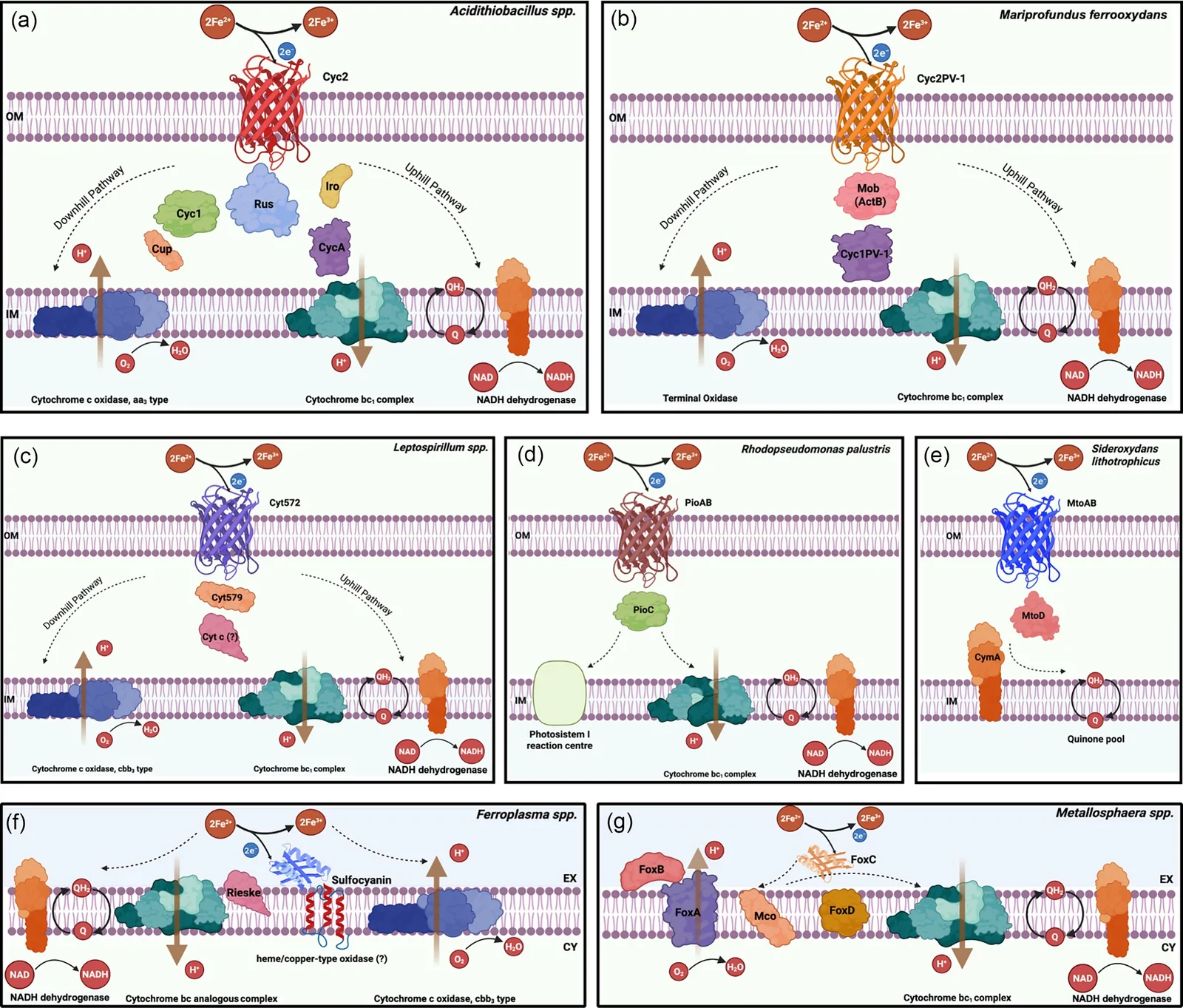
Schematic representation of the main iron oxidation pathways employed by different microbial groups. (a) *Acidithiobacillus* spp., (b) *Mariprofundus ferrooxydans*, (c) *Leptospirillum* spp., (d) *Rhodopseudomonas palustris*, (e) *Sideroxydans lithotrophicus*, (f) *Ferroplasma* spp., (g) *Metallosphaera* spp. OM, outer membrane; IM, inner membrane; CY, cytoplasm; and EX, extracellular.

The electron flow can be described as follows: Fe(II)→cyc2→rus→cyc1→cyt $a{{a}_3}{{O}_2}$ (Zhang S et al. 2018). All these proteins are encoded by the single-copy *rus* operon that includes the genes *cyc2→cyc1→cup→coxB→coxA→coxC→coxD→rus→ctaT→ctaB→ctaA* (Li L et al. 2023). The transcriptional activity of the operon is modulated by the nature of the electron donor substrate. High levels of expression are observed in cells grown with Fe²⁺ as the primary energy source, whereas in sulfur-grown cultures its expression is restricted mainly to the early exponential phase of growth (Yarzábal Andrés et al. 2004).

A second model for Fe(II) oxidation has been proposed in *Acidithiobacillus* spp. However, its physiological relevance remains controversial due to the lack of a complete and experimentally validated pathway (Zhan et al. 2019). This alternative route is hypothesized to involve an iron oxidase (Iro), a soluble high-potential iron–sulfur protein (HiPIP) localized in the periplasm. It is proposed to act as the initial electron acceptor in this putative downhill electron transfer chain (Ilbert and Bonnefoy 2013).

Not all *Acidithiobacillus* strains harbor the *iro* gene, and sequence variability among Iro homologs suggests functional diversification. Genomic and comparative analyses indicate that only a limited number of species within the genus, notably *A. ferrivorans, A. ferriphilus*, and *A. ferridurans possess iro*, whereas complete mechanistic models for this pathway are still lacking (Jones and Santini 2023). Additionally, two distinct classes of HiPIPs have been described within the genus *Acidithiobacillus*: Iro, encoded by the *iro* gene and associated with Fe(II) oxidation, and Hip, which has been implicated in electron transfer processes linked to sulfur metabolism (Zhang X et al. 2016).

Despite the uncertainty surrounding its role *in vivo*, Iro from *A. ferridurans* has been experimentally demonstrated to catalyze Fe(II) oxidation *in vitro*, supporting its potential function as an iron oxidase (Liu Y et al. 2023).

During Fe(II) oxidation, a minor fraction of electrons (~5%) is diverted into the reverse, or uphill, electron transfer pathway to generate reducing power in the form of NADH. In this route, electrons derived from Fe(II) are transferred sequentially through outer-membrane and periplasmic carriers, including Cyc2, rusticyanin, and CycA1, before entering the cytochrome bc₁ complex and the quinone pool, and finally reaching NAD⁺ via NADH dehydrogenase I (Fig. 1a) (Zhang S et al. 2018). In *A. ferrooxidans*, two distinct bc1 complexes have been identified. The complex encoded by the petI operon mediates the electron transfer when Fe²⁺ is available as a substrate, while the complex encoded by petII operon operates in the presence of sulfur as a substrate (Zhan et al. 2019).

### *Leptospirillum* spp

In *Leptospirillum* spp., the overall organization of the Fe(II)-oxidizing pathway is comparable to that described for *A. ferrooxidans*, although it relies on a distinct set of cytochromes and electron carriers specific to this genus (Levicán et al. 2012). More precisely, Fe(II) oxidation is mediated by the outer membrane c-type cytochrome Cyt572, which is proposed to function as the primary electron acceptor. Electrons are subsequently transferred to the periplasmic cytochrome Cyt579 as well as to an additional, yet uncharacterized, cytochrome c. From this point, electron flow can proceed along two alternative routes: either directly to a cbb₃-type cytochrome c oxidase in the inner membrane, where molecular oxygen is reduced, or through the cytochrome bc₁ complex and the quinone pool to the NADH dehydrogenase (complex I), thereby supporting the generation of reducing power through reverse electron transport (Fig. 1c) (Levicán et al. 2012, Ilbert and Bonnefoy 2013, Zhang X et al. 2016).

As discussed earlier, iron-oxidizing acidophilic archaea are mainly represented by the genera *Ferroplasma* and *Metallosphaera*. The iron oxidation routes operating in these organisms are distinct from each other and, notably, differ substantially from those identified in acidophilic bacteria, indicating that iron-based chemolithotrophy has likely arisen through separate evolutionary lineages (Dong et al. 2021).

### *Metallosphaera* spp

In *Metallosphaera*, ferrous iron oxidation is associated with the *fox* operon. In this system, Fe(II) is oxidized at the cell surface by the b-type cytochromes FoxCD. Electrons are subsequently conveyed via a multicopper oxidase (Mco) to the heme–copper oxidase complex FoxAB, where molecular oxygen is reduced, constituting the downhill electron transfer route. The reverse pathway is proposed to involve the same multicopper oxidase together with the cytochrome bc complex, the quinone/quinol pool, and NADH dehydrogenase (Fig. 1g) (Ilbert and Bonnefoy 2013, Dong et al. 2021).

### *Ferroplasma* spp

No homologs of the *fox* operon have been identified in members of the genus *Ferroplasma*. Instead, Castelle CJ et al. (2015) reported the isolation of an ~850 kDa membrane-associated protein complex that contains all the components required to support the complete pathway of Fe(II) oxidation coupled to oxygen reduction. Within this system, a blue copper protein called sulfocyanin, which is tightly associated with a fusion protein of heme/copper-type oxidase, directly interacts with the ferrous iron at the cell surface. In addition, a distinct protein complex of about 150 kDa was recovered from the membrane fraction and is proposed to correspond to a Rieske-type cytochrome b complex, likely responsible for transferring electrons from sulfocyanin to the quinone pool in the cytoplasmic membrane (Fig. 1f).

### Mariprofundus ferrooxydans

Concerning *Mariprofundus ferrooxydans* PV-1, Fe(II) oxidation is proposed to involve a Cyc2-like outer membrane iron oxidase together with the Mob protein (also referred to as ActB), a periplasmic Fe–S molybdopterin oxidoreductase that is thought to participate in the electron transfer (Li L et al. 2023). According to current models, electrons extracted from Fe(II) at the cell surface are transferred via an outer membrane cytochrome c (Cyc2PV-1) to a periplasmic cytochrome c (Cyc1PV-1) and subsequently delivered to terminal oxidases of currently uncertain identity, where molecular oxygen is reduced through the downhill pathway. In parallel, an alternative uphill electron transfer route has been proposed, in which electrons from Cyc1PV-1 are directed to the cytochrome bc1 complex, then to the quinone pool, and finally to NADH dehydrogenase, enabling the production of NADH (Fig. 1b). Proteomic analyses indicate a relatively low abundance of the bc1 complex under Fe(II)-oxidizing conditions, suggesting that the alternative complex III (ACIII), may partially substitute for this function (Barco et al. 2015, Li L et al. 2023).

### Sideroxydans lithotrophicus

In *Sideroxydans lithotrophicus*, the decaheme cytochrome MtoA, embedded within the outer-membrane protein MtoB, is proposed to catalyze Fe(II) oxidation and to transfer the released electrons to the inner membrane using a short cytochrome c protein MtoD and the membrane-anchored tetraheme cytochrome c CymA, ultimately feeding the quinone pool (Fig. 1e) (Liu J et al. 2012, Li L et al. 2023). In addition to the well-characterized iron oxidation pathway mediated by MtoA, Keffer and colleagues reported that the genome of *Sideroxydans lithotrophicus* encodes three distinct isoforms of the iron oxidase Cyc2, same protein that has been described for *Mariprofundus ferrooxydans* (Keffer et al. 2025). The authors proposed that the ability of *S. lithotrophicus* to utilize both aqueous Fe(II) and solid-phase electron donors, such as magnetite, necessitates the presence of multiple iron oxidation pathways. Consistent with this hypothesis, proteomic analyses revealed that Cyc2 is upregulated under growth conditions with both dissolved iron and magnetite. In contrast, the MtoAB system shows the strongest increase in expression specifically during growth on magnetite, suggesting a preferential role of MtoAB in electron uptake from solid iron minerals, whereas Cyc2 may function more broadly in the oxidation of soluble Fe(II) (Keffer et al. 2025).

### Rhodopseudomonas palustris

A comparable mechanism operates in *Rhodopseudomonas palustris*, where phototrophic Fe(II) oxidation depends on the *pioABC* operon. This operon encodes the decaheme cytochrome PioA, the outer-membrane protein PioB, and the periplasmic HiPIP PioC. PioA and PioB are homologous to MtoA and MtoB, respectively, and are thought to mediate electron transfer from extracellular Fe(II) to the periplasm. The electrons are then conveyed to PioC and subsequently delivered either to the photosynthetic reaction center or to the cytochrome bc₁ complex (Fig. 1d) (Ilbert and Bonnefoy 2013, Trindade et al. 2023).

In the following section, attention will be focused on the proteins that directly mediate ferrous iron oxidation.

## Ferrous iron–oxidizing proteins

Before describing the individual proteins, we note that the evidence for a direct role in Fe(II) oxidation differs considerably among the systems discussed below. To help the reader navigate this heterogeneity, Supplementary Table S1 summarizes all the proteins and protein complexes covered in this review, together with their functional category (iron-oxidizing, electron-transfer, or metabolic pathway-associated protein) and the level of evidence supporting their involvement in iron oxidation. In addition, the structures shown in this section are computational predictions and could not be validated against experimental data within the scope of this review; they are therefore intended to illustrate the systems discussed and to generate testable hypotheses, not to prove the mechanisms proposed. The full details of how they were obtained, including modeling parameters, confidence metrics and cofactor placement procedures, can be found in the Supplementary Materials (Supplementary Methods, Figs S1–S5 and Tables S2–S6).

### Cyc2

Cyc2 is recognized as the primary Fe(II)-oxidizing protein in *Acidithiobacillus ferrooxidans*. The protein is composed of 485 amino acid residues and has an estimated molecular mass of 52.4 kDa (Uniprot: B7JAQ7_ACIF2).

The N-terminal region contains a 31-amino acid signal peptide and a conserved c-type cytochrome motif (CXXCH), in which the heme group is covalently attached to the protein through two thioether bonds involving the cysteines and is coordinated by the histidine residue (Fig. 2) (Jiang et al. 2021). Structurally, this prosthetic group is an iron-containing porphyrin complex that underpins the electron-transfer function of cytochromes (Ilbert and Bonnefoy 2013).

**Figure 2:**
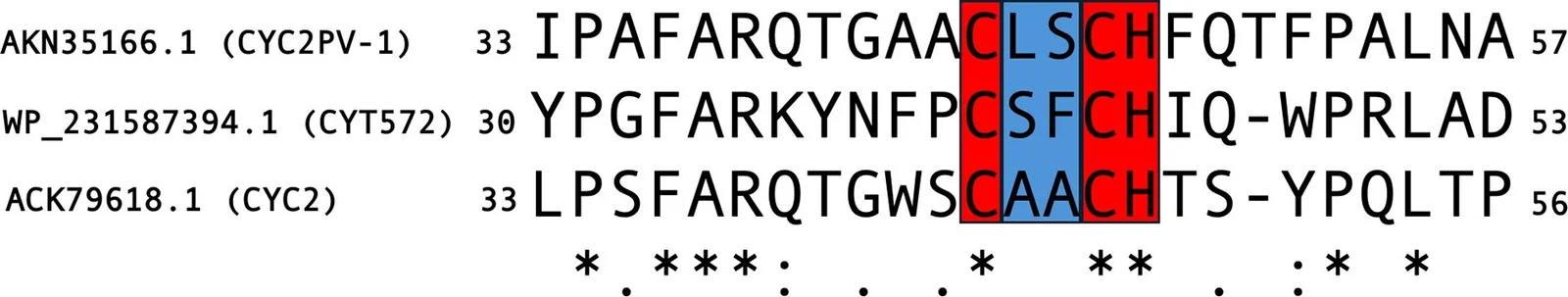
Multiple sequence alignment of the primary sequence of the cytochrome domain from three monoheme cytochromes c: AKN35166.1 (CYC2PV-1), WP_231587394.1 (CYT572), and ACK79618.1 (CYC2). The heme-binding motif is highlighted; conserved residues are marked with an asterisk (*) and variable residues are left unmarked.

The Cyc2 structure has not been experimentally resolved and was predicted by Jiang et al. (2021) using Rosetta protocol optimized for transmembrane beta barrel proteins under acidic conditions. The proposed model corresponds to a monomeric transmembrane β-barrel composed of 16 β-strands. This topology resembles that of porins, as shown by the superimposition with porin O of *Pseudomonas aeruginosa* (PDB 4RJX) structure that according to SWISS-MODEL (Waterhouse et al. 2018) has the higher coverage in terms of structure (i.e. 68%) (Fig. 3a) despite a low sequence identity (i.e. 11.78%). At the N-terminus, the β-barrel is fused to a cytochrome-like domain formed by several short α-helices and associated with a heme prosthetic group. The structure features a ring-shaped channel at its apical region oriented toward the periplasmic compartment, while flexible loop regions are exposed to the acidic extracellular environment and the N-terminal cytochrome-like domain extends into the periplasm (Jiang et al. 2021). Figure 3c shows the structure predicted by AlphaFold 3 (Abramson et al. 2024), including the heme cofactor, which is oriented toward the inside of the barrel. The SWISS-MODEL template covers only the β-barrel and not the N-terminal cytochrome domain (modeled residues 92–485 of 485), so the cofactor could not be placed by homology and the AlphaFold 3 model was used instead. The model is predicted with good overall confidence (mean pLDDT 79.9; 80.6 around the heme), with a single predicted axial ligand (His47) (Fig. S1, Table S2); the heme position and the putative Fe(II)-binding region should be regarded as predictions.

**Figure 3:**
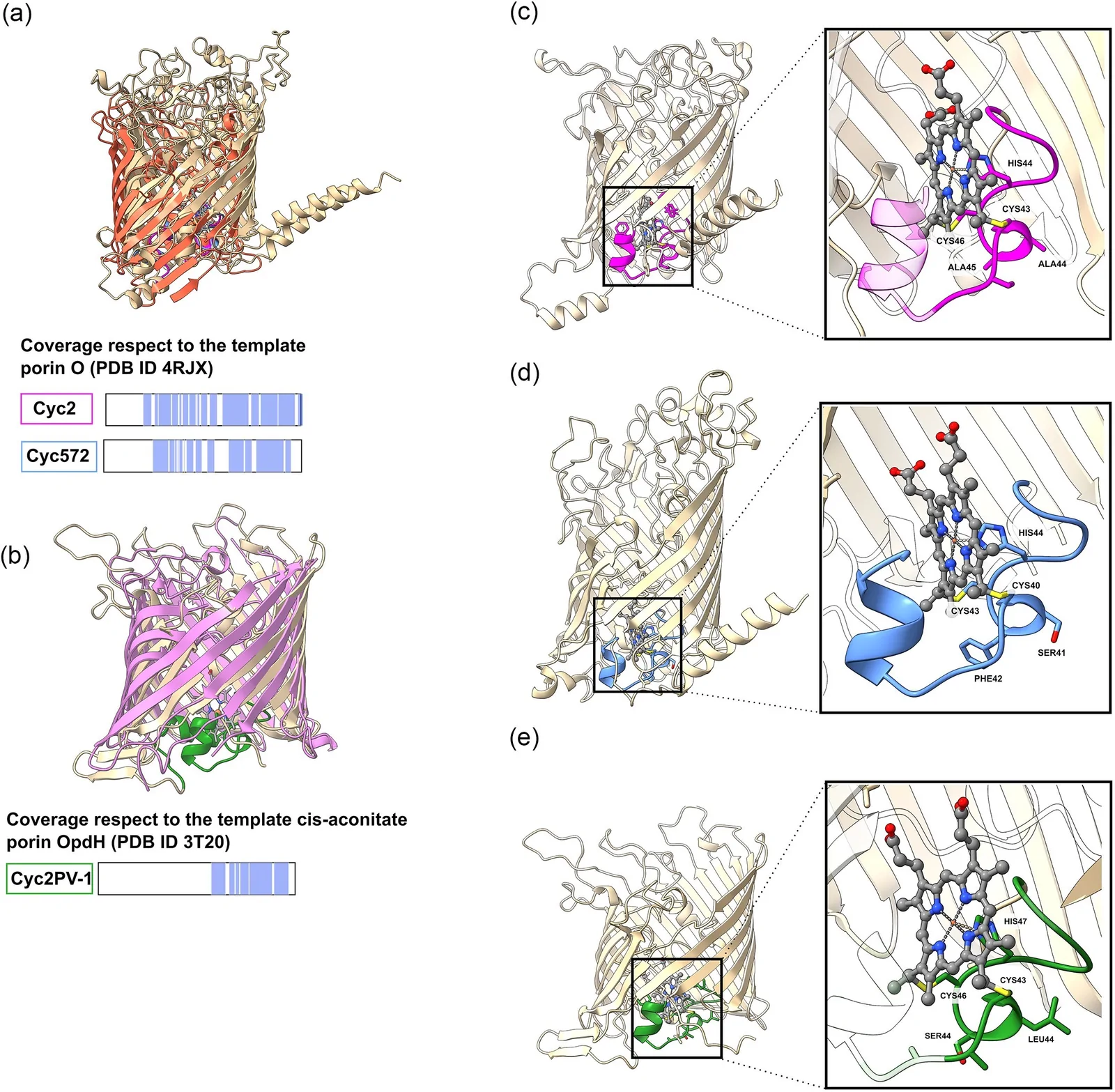
Monoheme cytochrome c proteins involved in ferrous iron oxidation. (a) Superimposition and coverage (68% for cyc2 and 51% for cyc572) of Cyc2, Cyc572, and the porin O [PDB ID: 4RJX (Modi et al. (2015))]. (b) Superimposition and coverage (35%) of Cyc2PV-1 the cos-aconitate porin OpdH (PDB ID: 3T20 [(Eren et al. 2012)]). (c) Cyc2, full protein is colored in neutral tint, while the cytochrome domain is marked with a more vivid one and presented also in the inset. In the cytochrome domain focus, amino acids belonging to the heme-binding motif (CAACH) are highlighted using sticks. (d) Cyt572, full protein is in neutral tint while the cytochrome domain is represented with a more vivid color. This domain is presented in the inset amino acids that bind the heme prosthetic group (CSFCH) are represented as sticks. (e) Cyc2PV-1, full structure in neutral tint, cytochrome domain is represented with a more vivid color; inset highlights CLSCH heme-binding residues, shown in sticks. Predicted structures, model source, confidence metrics, and cofactor placement are in the Supplementary Methods (Fig. S1, Table S2).

Castelle, Yarzábal, and colleagues were the first to experimentally characterize Cyc2 from *A. ferroxidans*. Using outer membrane-enriched fractions, they identified Cyc2 and demostrated its localization in the outer membrane, with portions of the protein exposed to the extracellular environment (Yarzábal A. et al. 2002). Subsequent work by Castelle C et al. (2008) further purified Cyc2 from solubilized membranes and confirmed its biochemical properties using EPR spectroscopy and mass spectrometry approaches. The EPR spectrum of the purified fraction supported the redox activity of Cyc2, showing its reduction upon exposure of Fe(II),consistent with its proposed role in iron oxidation. This behavior was accompanied by a relatively high redox potential of purified Cyc2 (560 mV at pH 4.8). In both studies, UV–Vis spectroscopy enabled the identification of spectral peaks corresponding to the γ, β, and α bands (420, 522, and 552 nm, respectively), characteristic of c-type cytochromes.

Jiang et al. (2021) investigated Cyc2 using computational approaches and, based on homology analyses, proposed the presence of two potential electron binding sites within the transmembrane β-barrel. Su et al. (2025) experimentally assessed these predictions by applying site-directed mutagenesis to disrupt the putative metal-binding residues, thereby validating the proposed extracellular electron transfer pathway. By integrating the monoheme redox-active protein Cyc2 into a supported lipid bilayer (SLB) platform, the overall electron transfer pathway of Cyc2, was elucidated. The first binding site, oriented toward the extracellular side of the β-barrel, is formed by residues Y262 and D308, whereas the second site is in the central region of the β-barrel and involves residues D137 and D138. Only through these sequential bindings electrons can be transferred to the heme group, possibly when inserted inside the barrel, and then consequently in the periplasm, to downstream acceptors in the iron oxidation pathway.

### Cyt572

The protein consists of 570 amino acids (61.3 kDa) and contains a single CXXCH heme-binding motif characteristic of c-type cytochromes (Uniprot: K4ENW4). N-terminal sequencing of the purified protein by Edman degradation yielded the first ten residues (YPGFARKYNF), which exactly matched the translated sequence of gene 630–6 that encodes for the protein. This sequence is preceded by a signal peptide, as predicted by the SignalP program, with a signal peptidase I cleavage site located between Ala and Tyr in the motif AANA/YPGF (Jeans et al. 2008).

Jeans et al. (2008) reported the identification and purification of a prominent cytochrome from *Leptospirillum* group II (LeptoII) biofilms collected at the Richmond Mine (USA). Cyt572 was purified from membrane fractions and characterized spectroscopy analysis. The reduced form of Cyt572 remained stable against oxidation for extended periods under atmospheric oxygen. The spectral and redox properties of Cyt572 were strongly modulated by pH, with acidic conditions inducing marked changes in its absorption features and oxidation state. These spectral features were independent of buffer composition, as identical responses to oxidation and reduction were recorded at the same pH in different buffer systems. Collectively, these findings indicate that the heme environment of Cyt572 is strongly influenced by pH, a property that may be functionally relevant for its biological role.

Notably, Cyc2 from *Acidithiobacillus ferrooxidans* and *Mariprofundus ferrooxydans*, Cyt572 from *Leptospirillum spp*. and PioB/MtoB from neutrophilic bacteria all share a porin-like β-barrel fold. For Cyt572, the best template according to SWISS-MODEL is also the porin O of *Pseudomonas aeruginosa*, in the 16-stranded β-barrel architecture, with a coverage of 51% and sequence identity 8.77%. As for Cyc2, the template does not include the N-terminal cytochrome domain (modeled residues 136–516 of 556) and no homology model was built (Fig. 3a). In 2023, Li L et al. (2023) reported a structural comparison between these proteins yielded pairwise TM-align scores between 0.50 and 0.74, values indicative of a conserved overall fold despite the very low sequence identity observed (6%–16%). The AlphaFold 3 prediction, including the heme cofactor, is reported in Fig. 3d (moderate overall confidence, mean pLDDT 74.0; 77 around the heme, with His44 as the only predicted axial ligand; Fig. S1, Table S2); the heme position remains a prediction. The structure is characterized by short turns connecting the strands and by extended inter-strand loops exposed on the extracellular side.

### Cyc2PV-1

*Mariprofundus ferrooxydans* dominates biomineralization processes in iron-rich marine hydrothermal ecosystems. Given its ecological significance, Barco et al. (2015) conducted a proteomic analysis of this microorganism to elucidate its physiology, focusing on electron transport chain proteins. An abundant outer membrane cytochrome c, termed Cyc2PV-1 (UniProt: A0A0H3ZGZ2_9PROT) emerged as a key candidate for initial Fe(II) oxidation. This protein was classified as a distant homolog of Cyc2, the iron-oxidizing cytochrome c previously characterized in *Acidithiobacillus ferrooxidans*. Sequence alignment between the two proteins (Cyc2 and Cyc2PV-1) revealed a highly conserved N-terminal region, sharing 51% sequence identity and 71% similarity, as well as preservation of the canonical heme-binding motif (CXXCH), despite relatively low overall sequence identity (20%) and similarity (42%) (Fig. 2). Likewise, Keffer et al. (2021) performed analyses of primary and secondary structure on Cyc2PV-1, identifying a typical N-terminal signal sequence alongside a lone CXXCH heme-binding site. Again, attached to this cytochrome region lies a porin domain structured as a β-barrel, featuring the 16 β-strands architecture. In this case, the template with the major coverage is Cis-aconitate porin OpdH of *Pseudomonas aeruginosa* (PDB 3T20 (Eren et al. 2012) with a coverage of 35% and identity of 14.38% (Fig. 3b). This template covers only part of the β-barrel (modeled residues 248–416 of 439) and not the heme-binding region, so the heme position can only be inferred from the AlphaFold 3 prediction. According to this structural organization, and the structure predicted by AlphaFold 3, which includes the heme (Fig. 3e; mean pLDDT 81.2; His47 as the only predicted axial ligand; Fig. S1, Table S2), the cytochrome domain may be positioned on the periplasmic side of the β-barrel. While the Cyc2 protein from *Acidithiobacillus ferrooxidans* had previously been characterized in its native host, neutrophilic IOB are difficult to cultivate in sufficient quantities for biochemical assays. Consequently, a heterologous expression strategy in *Escherichia coli* was adopted (Keffer et al. 2021). This approach is particularly relevant for advancing functional studies and for potential biotechnological applications, given that *E. coli* is the standard model organism for recombinant protein production. Spectroscopic analyses verified the redox capability of the recombinant protein, demonstrating that Cyc2-PV1 could be reduced by sodium dithionite. Moreover, incubation with Fe(II) induced the same spectral changes observed upon chemical reduction, indicating that the protein can accept electrons from ferrous iron and thus functions as an iron oxidase. The redox potential of Cyc2PV-1 was determined to be 208 ± 20 mV.

### Sulfocyanin

Available literature concerning the blue copper protein sulfocyanin (Uniprot: A0A1V0N1W7_9ARCH) from bacteria belonging to the genus *Ferroplasma* remains relatively limited. In contrast to the proteins discussed previously, this protein is characterized by the presence of copper as its metal cofactor, which constitutes a notable functional distinction. During electron transfer, the copper center undergoes a redox transition from the diamagnetic Cu(I) state to the paramagnetic Cu(II) state (Kanbi et al. 2002). Another relevant difference lies in its cellular localization, as sulfocyanin appears to be associated with the outer membrane through oxidase subunit II where it can directly interact with the substrate at the cell surface.

Sulfocyanin from *Ferroplasma acidarmanus*, rusticyanin from *Acidithiobacillus ferrooxidans* and the multicopper oxidase (Mco) from *Metallosphaera* species represent a clear example of structural similarity in terms of three-dimensional folding, as displayed by the SWISS-MODEL homology model of sulfocyanin, built on rusticyanin (PDB 1RCY; coverage 75%, sequence identity 22%) and reported in Fig. 4. Indeed, these proteins share a conserved structural framework composed of a central β-barrel or β-sandwich core with two mixed β-sheet regions. Despite exhibiting low sequence identity (~11%–14%), these proteins retain critical amino acid residues responsible for copper ion coordination, as described by Li et al. In rusticyanin, the coordinating residues follow the sequence His85, Ser86, Cys138, His143, and Met148, whereas in sulfocyanin a substitution of Ser86 with Thr114 is observed (Fig. 4) (Li L et al. 2023). The latter residues play a pivotal role in preserving protein stability under acidic conditions and in fine-tuning the redox potential, primarily through hydrogen-bonding interactions that modulate the electronic environment of the copper-binding site. In the model, the copper ion is transferred from the template; the overall model quality is modest (GMQE 0.41, QMEAN4 *z*-score −4.8), although the local quality at the copper site is higher (QMEANDisCo 0.57–0.66; Fig. S2, Table S3). The model therefore supports the conservation of the copper site, but does not allow conclusions on redox tuning.

**Figure 4:**
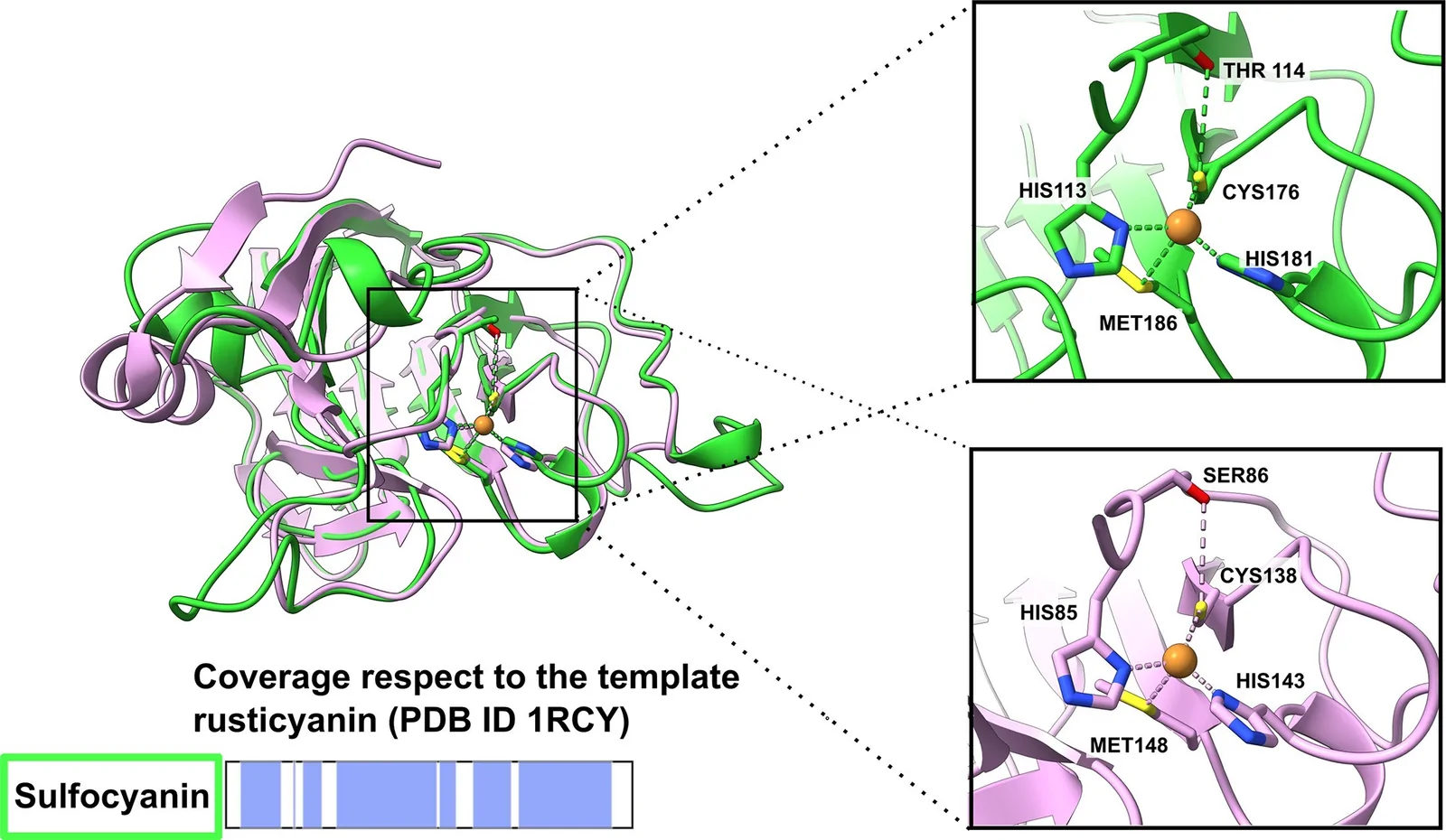
Copper protein invelved in iron oxidation pathways. Superimposition and coverage (75%) of sulfocyanin (in green, modeled by SWISS-MODEL) and rusticyanin, used as a template [in pink, PDB ID 1RCY (Walter et al. 1996)]. On the right, the detail of the two copper centers. Predicted structures, model source, confidence metrics, and cofactor placement are in the Supplementary Methods (Fig. S2, Table S3).

Starting from membrane fractions of *Ferroplasma acidiphilum*, Castelle CJ et al. (2015) purified two main protein complexes; a highly active assembly of ~850 kDa and a smaller, less active form of about 370 kDa. This protein complexes were further characterized by biochemical and proteomic analyses, which enabled the identification of sulfocyanin and several oxidase subunits, consistent with a putative respiratory electron transfer complex. Notably, iron oxidase activity colocalized with cytochrome c oxidase, supporting a coupled Fe²⁺-to-O₂ electron transfer pathway. Functional assays further confirmed that Fe(II)-oxidizing activity was markedly higher than that observed with cytochrome *c* as an artificial electron donor, indicating that Fe²⁺ is the native substrate. Measurements of oxygen consumption revealed high catalytic efficiency, demonstrating effective coupling between ferrous iron oxidation and oxygen reduction. Inhibition experiments showed that cytochrome c oxidase activity was almost completely suppressed by potassium cyanide (≈95% inhibition), in contrast, Fe(II)-oxidizing activity was only partially inhibited (≈40%–45%), indicating that sulfocyanin-mediated Fe²⁺ oxidation occurs upstream of the terminal oxidase reaction within the electron transfer chain.

### FoxC

In *Metallosphaera* species, FoxC (Uniprot: A4YE03_METS5, 548aa, ∼59 kDa) is a high-potential redox cytochrome b subunit within the fox gene cluster, proposed as the primary site for direct Fe(II) oxidation. Counts and colleagues identified FoxC within membrane fractions of *Metallosphaera sedula* using proteomic approaches. However, they were unable to isolate the protein through conventional biochemical methods, likely due to its extensive hydrophobic surface or the probable presence of substantial glycosylation, as reported for other proteins of the Crenarchaeota (Counts et al. 2022). The main structural difference between the previously described cytochromes c and the cytochrome b FoxC lies in the mode of heme binding. In cytochromes c, the heme group is covalently attached to the polypeptide chain through two thioether bonds, whereas in cytochromes b, the heme is noncovalently bound (Ilbert and Bonnefoy 2013). FoxC is functionally coupled to FoxD, a subunit with 10 predicted transmembrane helices, and together they constitute a membrane-associated complex that directs electrons from Fe(II) oxidation toward the respiratory electron transport system. This is demonstrated by strong transcriptional upregulation of both genes under Fe(II)-growth conditions and comparative genomics across iron-oxidizing *Sulfolobaceae* (Kozubal et al. 2011). Structural modeling provides only tentative support for this functional assignment. As highlighted by Kozubal et al., FoxC presents a conserved sub-domain within the heme binding domain of the γ-subunit of ethylbenzene dehydrogenase [PDB 2IVF (Kloer et al. 2006)]. Despite homology modeling by SWISS-MODEL did not allow the complete reconstruction of the protein (coverage of 10%, Fig. 5a), a sequence identity of 30% was identified in this region. Neverthless, we reported an AlphaFold 3 generated structure having a very well aligned domain of the γ-subunit (Fig. 5b). The FoxC model is predicted with good confidence (mean pLDDT 84.3; 94 around the heme; Fig. S3, Table S4). This reference protein adopts a characteristic crescent-like fold in which a noncovalently bound heme b group is tightly embedded within the protein scaffold (Li L et al. 2023). Interestingly, the modeled FoxC structure is predicted to coordinate the heme iron through lysine 478 and methionine 296, although the Lys478–Fe distance in the FoxC–FoxD model (1.46 Å) is shorter than expected for a Fe–N bond and this ligation should be regarded as tentative; this latter methionine was not found by sequence alignment by Kozubal et al., who proposed different residues (e.g. Arginines) to coordinate such residues. Furthermore, this fact was connected to the higher redox potential of FoxC, compared to that of CbsA (+400 mV, measured experimentally), a cytochrome b found in several members of the *Sulfolobales*, and the fact that *F*oxC is active at lower pH values (Kozubal et al. 2011).

**Figure 5:**
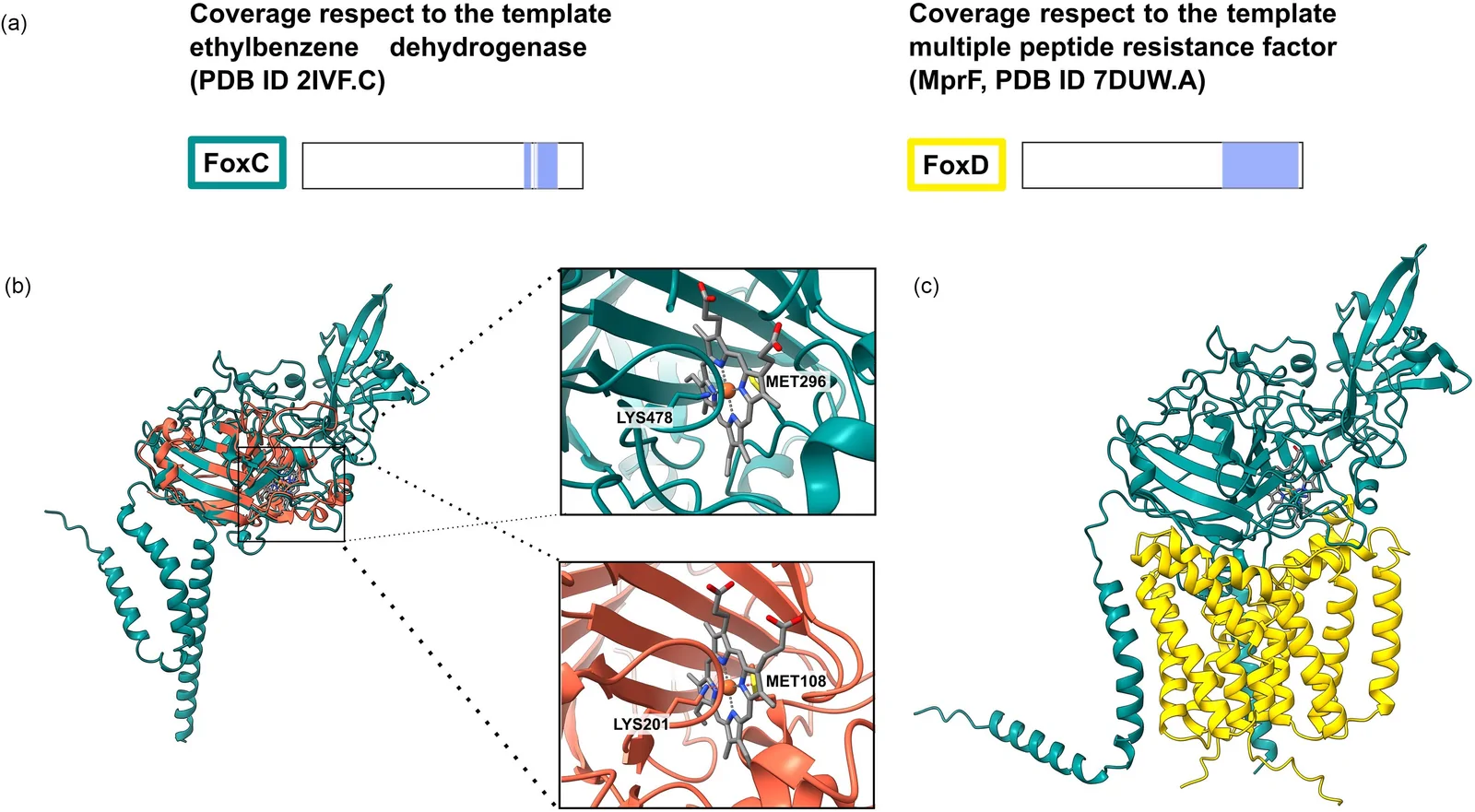
FoxC–FoxD complex of Metallosphaera sedula. (a) Coverage (∼10% for FoxC and ∼25% for FoxD) by SWISS-MODEL. (b) Prediction by AphaFold 3 of FoxC superimposed to the ethylbenzene dehydrogenase (that overlap only the central core of FoxC), subunit gamma [PDB ID 2IVF (Kloer et al. 2006)]. The highly conserved region corresponds to the domain surrounding the binding site of the heme group. In the inset are highlighted the Lysines and Methionines coordinating the heme molecule, FoxC in the top and ethylbenzene dehydrogenase on the bottom. (c) FoxC-FoxD complex predicted by Alphafold3, N- and C-terminal region of FoxC well adapt to the binding of FoxD. Predicted structures, model source, confidence metrics, and cofactor placement are in the Supplementary Methods (Fig. S3, Table S4).

Previous work indicates that the predicted three-dimensional structure of FoxD (UniProt A4YE02) appears to be homologous to chain A of nitric oxide reductase from *Geobacillus stearothermophilus* [PDB 3AYF (Matsumoto et al. 2012)]. In contrast, using SwissModel, the template with the highest coverage is the multiple peptide resistance factor (MprF) of *Martinezella tropici*, which is insufficient for modeling the FoxD structure due to low coverage (27% coverage, 16% sequence identity; Fig. 5a). Moreover, SwissModel is unable to generate a homology model using chain A of PDB 3AYF as a template. Nevertheless, by modeling the FoxC–FoxD complex using AlphaFold3 (Fig. 5c), it is interesting to note that co-predicted FoxC C- and N-term are well adapted to the binding of FoxD. The complex is predicted with moderate confidence (ipTM 0.77; mean interface PAE 12.7 Å; Fig. S3, Table S4), and this observation should be regarded as a hypothesis on the FoxC–FoxD interaction rather than as evidence for it.

### PioA

PioA is a decaheme c-type cytochrome composed of 540 amino acids, with an approximate molecular mass of 40 kDa (UniProt: A1EBT2_RHOPT). The pioA gene (Rpal_0817) from *Rhodopseudomonas palustris* encodes a protein containing a canonical signal peptide (residues 1–40), indicating periplasmic targeting. The C-terminal region includes a hydrophobic segment (residues 241–263), followed by ten CXXCH motifs responsible for covalent heme attachment.

Jiao and colleagues employed experimental approaches to identify the pioABC operon (Jiao and Newman 2007). Cells of *Rhodopseudomonas palustris* grown in the presence of Fe(II) allowed purification of a soluble membrane-associated decaheme cytochrome c with the biochemical properties of PioA. Deletion mutants lacking the entire *pioABC* operon exhibited an almost complete loss of Fe(II)-oxidizing capacity, demonstrating the essential role of this gene cluster in iron oxidation. A similar phenotype was observed in Δ*pioA* mutants, indicating that PioA acts as the direct Fe(II) oxidase. A distinctive feature of this protein is its cellular localization: whereas previously characterized iron-oxidizing cytochromes were typically located in the outer membrane or exposed to the extracellular environment, PioA was found in the soluble membrane fraction, consistent with a periplasmic localization.

Deletion mutant production further demonstrated that PioA activity is strictly dependent on PioB (Uniprot: A1EBT3_RHOPT), the subunit responsible for anchoring PioA to the outer membrane. In Δ*pioB* mutants, PioA was detected exclusively in a soluble form, yet these strains were unable to oxidize Fe(II) in cell suspension assays. Conversely, in Δ*pioA* mutants, PioB could not be detected in the outer membrane, indicating a mutual dependence of the two proteins for correct subcellular localization. Collectively, these results strongly suggest that Fe(II) oxidation does not occur in the periplasm, where ferric iron precipitation would pose a severe physiological problem, but rather at the extracellular face of the outer membrane. Moreover, they indicate that oxidative activity requires formation of the PioAB complex and that soluble periplasmic PioA alone is not functionally sufficient.

PioA possesses an unusually large N-terminal extension of ~200 amino acids that is absent in other smilar decaheme cytochromes such as MtrA from *Shewanella oneidensis* and MtoA from *Sideroxydans lithotrophicus*. In Fig. 6a, the superimposition of the PioA–PioB and MtoA–MtoB complexes predicted with AlphaFold 3 over the Mtr complex is reported. SWISS-MODEL could model the single chains using the Mtr complex as template (coverage 48% and 75% for PioA and MtoA, 80% and 83% for PioB and MtoB) but neither the hemes nor the interface between subunits, and the complexes were therefore predicted with AlphaFold 3, including 10 heme cofactors on chain A. The long N-terminal region is conserved among PioA homologs from diverse phototrophic bacteria but it does not contain any recognizable functional domains. In the AlphaFold 3 model of the PioA–PioB complex, PioA is predicted with lower confidence than the other proteins (mean pLDDT 58): the N-terminal extension is largely below pLDDT 50 (47% of PioA residues), which is compatible with a largely unstructured region, although this cannot be established from the model alone, whereas the heme-binding region is predicted with higher confidence (pLDDT 77–90 around the hemes; Fig. S4, Table S5). Experimental evidence suggests that this region plays a stabilizing and regulatory role rather than a direct catalytic function (Gupta et al. 2019). Structurally, PioA is largely dominated by flexible loop regions (60.6%), accompanied by a moderate proportion of α-helices (37.8%) and only a marginal contribution from β-sheet elements (1.6%) (Fig. 6b, c) (Li L et al. 2023).

**Figure 6:**
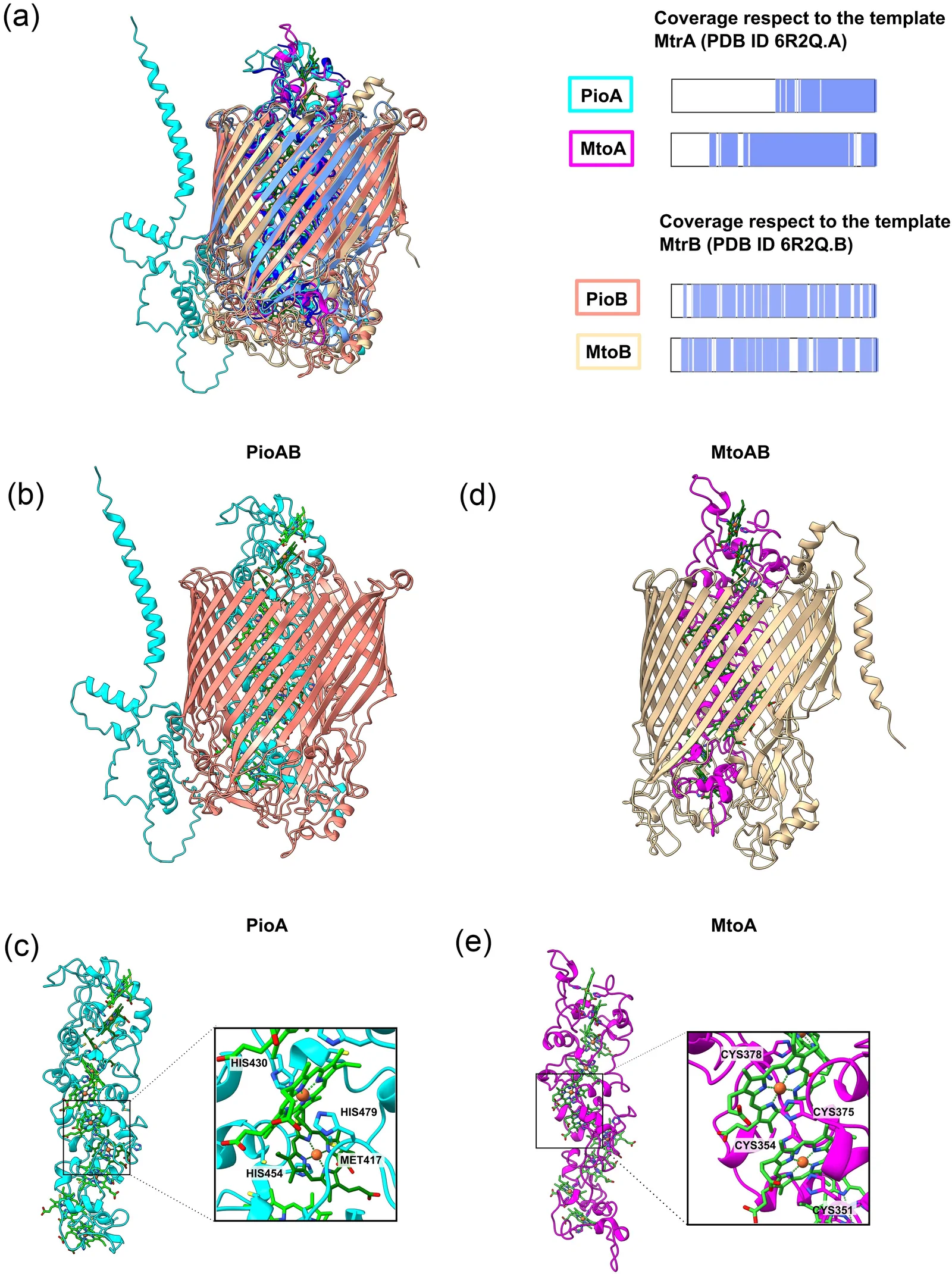
Structural organization of two decaheme cytochromes involved in iron oxidation. (a) Superimposition of the modeled PioA/PioB complex (cyan and salmon) and MtoA/MtoB complex (magenta and beige) over the reference Mtr complex [PDB ID: 6R2Q (Edwards et al. 2020)]. Folded structures were obtained with AlphaFold 3 only when the A and B subunits were predicted together. Sequence coverage of SWISS-MODEL homology models is also reported; however, the corresponding structures are not shown due to the absence of heme groups. (b) The decaheme cytochrome PioA is shown in complex with its membrane-anchoring partner protein PioB, which is represented in salmon. (c) Isolated cytochrome PioA, depicted in cyan, with two selected heme groups highlighted in the inset, with key residues represented as sticks. In dark green, the heme group coordinated by His/Met can be observed, whereas in light green the heme group coordinated by His/His is shown. (d) Decaheme cytochrome MtoA in magenta, in complex with its membrane-anchoring partner protein MtoB, wich is in beige. (e) Isolated cytochrome MtoA is depicted in magenta, with two selected heme groups highlighted in green in the inset, key residues represented as sticks, similar to panel c. Predicted structures, model source, confidence metrics, and cofactor placement are in the Supplementary Methods (Fig. S4, Table S5).

Gupta et al. (2019) expressed PioA heterologously in *Escherichia coli* and also purified the protein from the native host for biochemical characterization. Upon reduction with dithionite, PioA showed spectral features typical of reduced c-type cytochromes (Soret at 418 nm with α/β bands at 523 and 551 nm). Fe(III) caused reoxidation, while Fe(II) reduced PioA only at alkaline pH (9–10), indicating pH-dependent electron uptake.

In contrast, PioC, a high-potential iron–sulfur protein (HiPIP), functions as an electron shuttle between the PioAB complex and the photosynthetic reaction center. However, Trindade and colleagues demonstrated that PioA can also transfer electrons directly to the light-harvesting–reaction center (LH–RC) (Trindade et al. 2023). This alternative pathway provides a low-efficiency auxiliary route for electron transfer and explains the residual photoferrotrophic activity observed in the absence of PioC.

Using spectroelectrochemistry and cyclic voltammetry, Li DB et al. (2020) showed that PioA is redox-active between approximately −400 and +250 mV. In addition, they identified the presence of noncanonical axial ligands coordinating certain heme groups, in contrast to the classical His/His coordination typical of c-type cytochromes. By comparing the absorption spectra of PioA under air-equilibrated, ascorbate-reduced, and dithionite-reduced conditions, they identified hemes 2, 3, and 7 as likely candidates for His/Met coordination, as shown in modeled structure in Fig. 6c. In the AlphaFold 3 model of the PioA–PioB complex, hemes 2, 3, and 7 (numbered from the N-terminus according to the CXXCH motifs) are indeed the only ones predicted with His/Met axial ligation, an observation compatible with, but not a validation of, this proposal (Fig. S4). This ligand configuration contributes to the relatively high redox potential of PioA compared to its homologs, such as MtoA from *Sideroxydans lithotrophicus* (Li DB et al. 2020).

### MtoA

MtoA (Uniprot: D5CMQ0) protein is composed of 317 amino acids, with a calculated molecular mass of ~33.6 kDa. The sequence contains 10 conserved CXXCH motifs, indicating that the mature protein can accommodate up to ten heme cofactors. Comparative sequence analysis showed that MtoA shares 39% amino acid identity with PioA, while MtoB (Uniprot: D5CMP9_SIDLE) exhibits 17% identity with PioB. In the AlphaFold 3 model of the MtoA–MtoB complex, MtoA is predicted with good confidence (mean pLDDT 79; 85% of its residues with pLDDT ≥ 50), and the 10 hemes, all with predicted His/His axial ligation, with local pLDDT of 85–94; the cofactor positions and the interface with MtoB (ipTM 0.87) remain predictions (Fig. S4, Table S5).

Liu J et al. (2012) heterologously expressed MtoA from *Sideroxydans Lithotrophicus* using *Shewanella oneidensis* MR-1 as the host organism and subsequently characterized the protein using standard biochemical approaches. In addition to the histidine residues involved in heme attachment, MtoA possesses fourteen additional histidines, most of which are likely to function as distal axial ligands for the heme iron.

Structural modeling predicts that PioB and MtoB adopt a porin-like architecture composed of 26 antiparallel β-strands enriched in hydrophobic residues, a feature consistent with the solubility and stability of the Pio/Mto complexes within the lipidic outer membrane (Fig. 6a). In the AlphaFold 3 models, PioB and MtoB are predicted with good confidence (mean pLDDT 82.7 and 85.3). The subunit interface has ipTM values of 0.86 (PioA–PioB) and 0.87 (MtoA–MtoB), but the mean interface PAE is higher for PioA–PioB (19.1 Å) than for MtoA–MtoB (12.1 Å), indicating a less well defined relative arrangement of the PioA–PioB subunits (Fig. S4, Table S5). β-barrel proteins are proposed to orient and anchor PioA/MtoA such that the heme chain is arranged perpendicular to the membrane plane, thereby optimizing electron transfer pathways (Fig. 6d) (Li L et al. 2023). Instead, MtoA exhibits a conformational organization that is predominantly characterized by flexible loop regions (64.3%), while α-helical content accounts for 34.2% and β-sheet structures are minimally represented (1.5%) (Li L et al. 2023), able to accomodate the ten heme cofactors (Fig. 6e). In the AlphaFold 3 models, the 10 hemes show predicted histidine axial ligation (10 His/His in MtoA; seven His/His, and three His/Met in PioA) with local pLDDT of 77–94 around the hemes; they are, however, predictions of the cofactor position and not experimental evidence (Fig. S4, Table S5).

The redox properties of MtoA were investigated by cyclic voltammetry over a range of pH values from 7 to 9. At all tested pH conditions, MtoA remained redox active, displaying midpoint potentials spanning approximately +100 to −400 mV vs. the standard hydrogen electrode. Furthermore, Liu J et al. (2012) demonstrated that MtoA exhibits ligand-dependent Fe(II) oxidation activity. Stopped-flow spectrophotometric analyses showed that extent of MtoA reduction at equilibrium depended strongly on the chemical form of Fe(II), following the order: Fe(II)Cl₂ > Fe(II)–citrate > Fe(II)–NTA > Fe(II)–EDTA. These findings suggest that the nature of the coordinating ligand significantly influences both the oxidation kinetics of Fe(II) complexes catalyzed by MtoA and the associated equilibrium constants.

### IRO

The Iro protein is a 6 kDa periplasmic high redox potential iron–sulfur protein (HiPIP) that is proposed to act as the primary electron acceptor in the alternative ferrous ion oxidation pathway of *Acidithiobacillus* species (Uniprot: P50500). HiPIPs are generally isolated from purple phototrophic bacteria and constitute a class of small proteins (6–10 kDa) containing a [Fe₄S₄] cluster. This cluster undergoes one-electron redox transitions between the [Fe₄S₄]²⁺ and [Fe₄S₄]³⁺ states (Zeng J et al. 2007).

Production and purification of Iro from distinct *Acidithiobacillus* species have been documented, both employing *E. coli* BL21 for protein expression. Liu Y et al. (2023) expressed the protein from *A. ferridurans* strain RIV 11, while earlier work focused on the Iro homolog from *A. ferrooxidans* Fe–1, with the two variants exhibiting 77.36% sequence identity (Zeng J et al. 2007). Characterization of Iro was performed using UV–Vis spectroscopy, revealing an absorption maximum at 388 nm. Electron paramagnetic resonance spectroscopy indicated a signal only in the oxidized state, consistent with the oxidation of the iron–sulfur cluster (Zeng J et al. 2007, Liu Y et al. 2023).

Circular dichroism (CD) indicated that both oxidized and reduced forms share similar secondary structure profiles, confirming that redox state does not affect protein folding. Similarly, FTIR spectroscopy revealed no structural changes between the two redox states. Furthermore, the optical spectrum of oxidized Iro displayed a notable increase in absorbance between 450 and 550 nm compared with the reduced protein, appearing more intense and reddish. Monitoring the absorbance change at 500 nm allowed determination of the second-order kinetic rate constant (4.6 × 10³ M⁻¹ s⁻¹) for electron transfer from ferrous ion to oxidized iron oxidase using a pseudo-first-order reaction model (Liu Y et al. 2023).

The first 37 amino acids of Iro constitute a Tat signal sequence, consistent with its periplasmic localization. The [Fe₄S₄] cluster is centrally positioned within the protein and is surrounded by several aromatic residues, which likely contribute to cluster stability (Fig. 7). In the SWISS-MODEL homology model built on the high-potential iron–sulfur protein of *Rhodocyclus tenuis* (PDB 1ISU; 44% sequence identity, 58% coverage corresponding to the mature protein), the cluster was positioned according to the template. Structural modeling and sequence alignment suggest that the cluster is coordinated by four highly conserved cysteine residues Cys20, Cys23, Cys32, and Cys45, with interatomic distances to the corresponding iron atoms of the cluster within 3 Å, comparable to previously reported HiPIP structures (Fig. 7). As the cluster and these distances are inherited from the template, they are not an independent prediction; the model is of modest global quality (GMQE 0.38, QMEAN4 *z*-score −2.8), although the cysteine environment is locally better defined (QMEANDisCo 0.63–0.72; Fig. S5, Table S6). The cluster is also enveloped by hydrophobic residues, among which Tyr10, Phe26, and Phe48 are essential for maintaining stability under acidic conditions (Fig. 7). Substitution of these aromatic residues with alanine or serine partially disrupts the microenvironment of the binding pocket, preventing cluster binding under acidic conditions. In contrast, replacement with alternative aromatic residues preserves cluster stability, as the aromatic groups shield the [Fe₄S₄] center from acidic hydrolysis (Zeng J et al. 2010).

**Figure 7:**
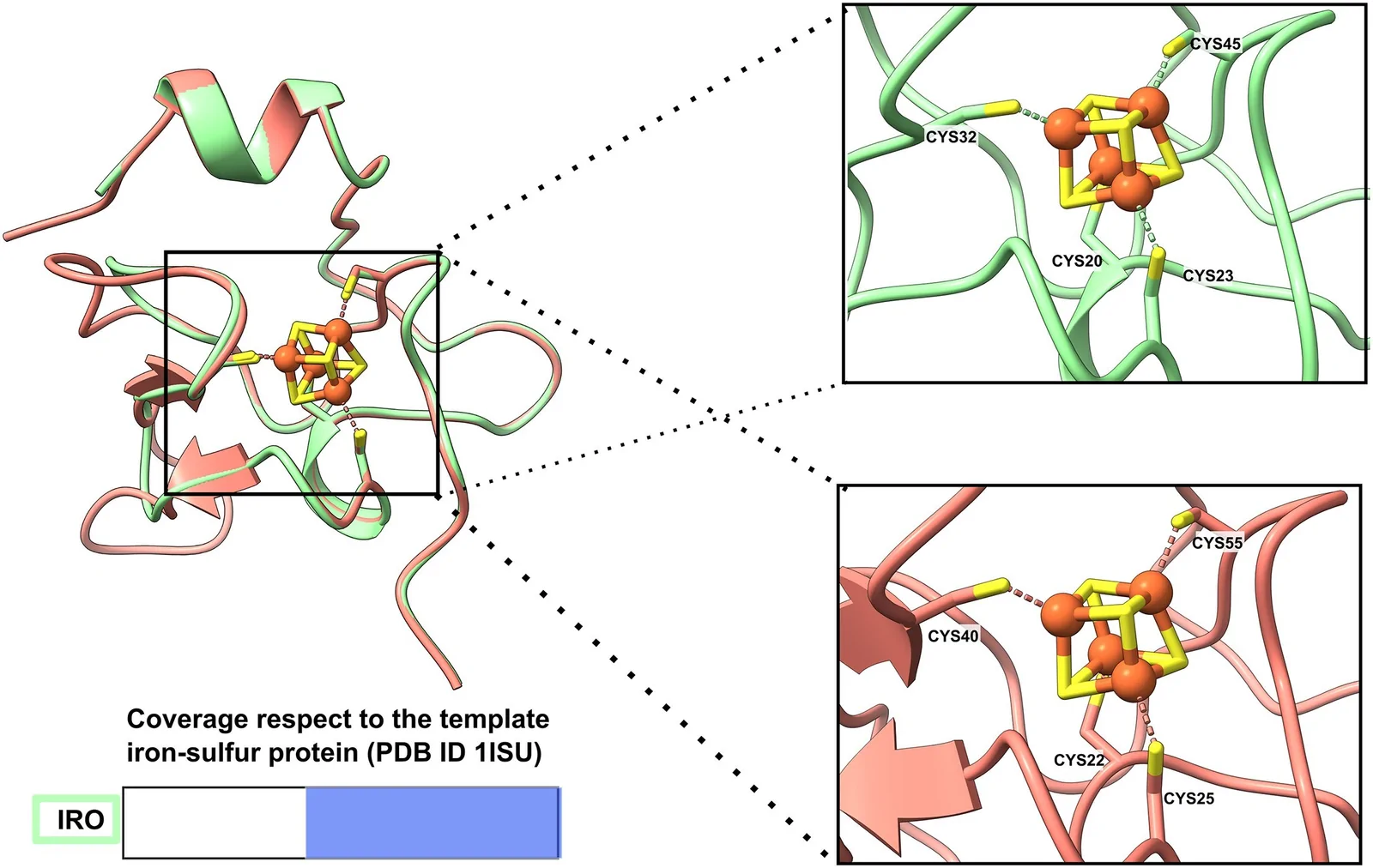
Iro protein of Acidithiobacillus ferrooxidans. Superimposition and coverage (58%) of Iro (modeled by Swiss Model) and iron–sulfur protein of Rhodocyclus tenuis, used as a template [PDB ID 1ISU (Rayment et al. 1992)]. On the right, the detail of the two [Fe₄S₄] clusters, Iro in the top, iron–sulfur protein of Rhodocyclus tenuis on the bottom. Predicted structures, model source, confidence metrics and cofactor placement are in the Supplementary Methods (Fig. S5, Table S6).

Molecular simulations performed by Liu Y et al. (2023) examined interactions between iron oxidase and ferrous ions under physiologically relevant conditions (aqueous solution, pH 2.0, 300 mM NaCl). The most frequently observed contact configuration was selected as a representative model, in which the ferrous hexahydrate complex ([Fe(H₂O)₆]²⁺) approaches a protein surface formed by residues Asn62, Ala84, and Ala86, directly connected to the [Fe₄S₄] cluster and representing the shortest pathway between the cluster and solvent.

## Computational approaches to study iron-oxidizing proteins

The experimental characterization of iron-oxidizing proteins is inherently challenging. These proteins are frequently membrane-associated, often unstable under standard laboratory conditions, and difficult to obtain in sufficient quantities for biochemical analysis. For these reasons, computational biology has emerged as a powerful complementary approach for investigating their structure, dynamics, and functional properties at the molecular level. Over the past two decades, a progressive hierarchy of methods has been applied to these proteins, ranging from static-structure prediction to quantum-mechanical simulations of electron transfer.

### Structural modeling

In the absence of experimentally determined three-dimensional structures, homology modeling and *ab initio* prediction methods have provided the first structural insights into iron-oxidizing proteins. The most notable example is Cyc2 from *A. ferrooxidans*, for which no crystal structure is currently available: an initial model based on sequence coevolution analysis was proposed by Abriata et al. and later refined by Jiang et al. (2021) using a Rosetta protocol optimized for transmembrane β-barrel proteins under acidic conditions (Abriata 2018, Jiang et al. 2021). This model was subsequently used to propose two Fe²⁺-binding sites, later validated by mutagenesis (Su et al. 2025). Analogous approaches were applied to Cyt572 from *Leptospirillum* spp. and to FoxC/FoxD from *Metallosphaera*, revealing architectural homologies with known porins and membrane-bound oxidoreductases (Li L et al. 2023). An earlier landmark in this tradition is the molecular modeling of the Iro protein from *Acidithiobacillus ferrooxidans* Fe-1, whose gene was cloned and expressed in *E. coli*: computational modeling of the recombinant protein identified the specific cysteine residues coordinating the [Fe₄S₄] cluster, representing one of the first direct applications of structure prediction to a ferrooxidant Fe–S protein (Zeng J et al. 2007).

More recently, the successive releases of AlphaFold2 (Jumper et al. 2021) and AlphaFold3 (Abramson et al. 2024) have expanded the available toolset for structural prediction of these proteins. While AlphaFold2 does not model prosthetic groups, a limitation partially addressed by the AlphaFill post-processing pipeline (Hekkelman et al. 2023), AlphaFold3 jointly predicts protein coordinates alongside metal ions and small molecules. For protein–protein complexes, AlphaFold-Multimer (Evans et al. 2021) and docking algorithms have been used to reconstruct the Cyc2–rusticyanin–Cyc1 assembly of *A. ferrooxidans*, identifying interface residues consistent with sequential electron transfer through the outer membrane (Patra et al. 2013, Jiang et al. 2021). A systematic benchmark on a dataset of 887 metalloproteins containing biologically relevant metals including iron demonstrated that AlphaFold3 achieves high confidence in metal ion placement, particularly for sites with three or more coordinating residues, outperforming dedicated tools such as Metal3D (Dürr and Rothlisberger 2024). Despite this progress, AlphaFold3 does not model the electronic structure or redox state of the metal center, and its predictions for unusual or rare coordination geometries, such as those encountered in high-potential [4Fe–4S] clusters, may still require QM/MM refinement to reach mechanistic accuracy. Complementary bioinformatics strategies remain therefore relevant for Fe–S protein identification at the proteome scale: penalized linear models combined with hidden Markov model-based sequence descriptors have enabled large-scale discovery of Fe–S cluster proteins in microbial genomes (Estellon et al. 2014), and chemoproteomic approaches have extended this inventory by monitoring cluster occupancy across entire *E. coli* proteomes *in vivo* (Bak and Weerapana 2024).

### Molecular simulations

Classical molecular dynamics (MD) simulations have been extensively used to investigate the conformational dynamics and stability of iron-oxidizing and iron–sulfur (Fe–S) cluster proteins. In rusticyanin, MD simulations of the Met148Leu variant revealed local destabilization of the copper-binding site, providing a structural explanation for the altered redox properties observed experimentally (Jafarpour et al. 2021). At the supramolecular level, MD simulations of the *A. ferrooxidans* respiratory complex identified key contacts between Phe312 of Cyc2 and Asp73 of rusticyanin, and between His143 of rusticyanin and Glu121 of Cyc1 (Patra et al. 2013). Beyond strict iron-oxidizing systems, traditional MD has been very insightful for related Fe–S cluster proteins: microsecond-scale simulations of human NEET proteins in oxidized and reduced states revealed allosteric communication between the loop L2 region and the cluster binding site (Zuo et al. 2021), while studies on bacterioferritin demonstrated that coupled thermal fluctuations in pore-lining residues facilitate cation passage during iron loading and release (Rui et al. 2012). In cytochrome P450 systems, MD trajectories have characterized how redox partner binding shifts the enzyme toward an open conformational state competent for proton relay during oxygen activation (Hollingsworth et al. 2016, Poulos and Follmer 2022), illustrating the general power of classical MD to capture redox-coupled conformational transitions inaccessible to static structural models.

Although parameter sets have been developed for canonical iron cofactors such as heme groups (Oda et al. 2005, Shahrokh et al. 2012) and [Fe–S] clusters (Carvalho and Swart 2014), these are system-specific and not transferable to non-standard coordination environments. Indeed, parameters for iron–sulfur clusters cannot be generalized, nor be easily automated, and hence should be obtained for each system separately (Hu and Ryde 2011). To the best of our knowledge, no validated force field parameters exist for the metallic centers of Cyc2 and Iro, whose geometry, charge distribution, and spin state require quantum mechanical treatment for reliable description. In general, parameters have traditionally been derived case by case using the Seminario method applied to DFT-optimized geometries (Carvalho et al. 2013), or alternatively by restraining metal–ligand distances to crystallographic values and treating the coordination environment as rigid (Horch et al. 2016) an approach that further precludes any description of redox-state changes.

Recent tools have substantially lowered this barrier. easyPARM (Abdelgawwad and Francés-Monerris 2025) and Metallicious (Piskorz et al. 2024) now automate the derivation of AMBER-compatible parameters for metal complexes of arbitrary coordination geometry directly from QM frequency calculations, making metalloprotein MD accessible without specialized expertise in force field development. A complementary avenue is offered by reactive force fields: a ReaxFF parametrization for Fe–S clusters in explicit water enables simulations in which iron–sulfur bond breaking and formation are explicitly modeled (Moerman et al. 2021). Nevertheless, no classical or reactive-classical framework can describe spin-state changes and long-range charge reorganization during electron transfer, and this boundary defines the domain where hybrid QM/MM methods become essential.

Hybrid QM/MM methods treat the metallic center at the DFT level while describing the surrounding protein classically, coupling the two regions through electrostatic embedding (Vidossich and Magistrato 2014). Benchmark studies on rubredoxin showed that electrostatic contributions from residues several ångströms from the iron center measurably shift the calculated redox potential, underscoring the need for an explicit protein environment (Sulpizi et al. 2007). For type 1 copper proteins, QM/MM analyses have shown that charged residues in the second coordination sphere directly tune the redox potential by modulating the electronic properties of the metal center (Bím and Alexandrova 2021), a finding with direct implications for understanding how sequence variation controls electron transfer rates in iron-oxidizing chains. More broadly, QM/MM simulations have been used to link atomic-level protein structure to measurable electron transfer rates, providing a mechanistic bridge between structural data and electrochemical observations (Zeng X et al. 2008, Teuffel et al. 2025). Despite these advances, the application of such methods to the complete electron transfer chain of iron-oxidizing organisms remains largely unexplored and represents one of the most compelling open questions in the computational biology of these systems.

### Computational prediction of redox potentials

The redox potential of each cofactor determines the directionality and efficiency of electron flow along the iron oxidation chain. Three main computational strategies are currently available, each offering a different balance between accuracy and computational cost (Jafari et al. 2022) and a fourth data-driven approach is rapidly emerging.

Physics-based quantum-chemical methods represent the most established framework. QM cluster models embedded in a continuum dielectric (COSMO) have been benchmarked on twelve iron–sulfur clusters spanning six structural types, including [4Fe–4S] HiPIPs; the best-performing protocol achieved a mean absolute deviation of 0.17 V using TPSS/def2-SV(P) on a ∼300-atom QM region (Jafari et al. 2022). An analogous benchmark for type 1 blue copper proteins explicitly included rusticyanin from *A. ferrooxidans* in both wild-type and Met148 mutant forms: the optimal method reproduced the exceptionally high redox potential of rusticyanin (+667 mV) and its mutation-dependent variations with a mean absolute deviation of 0.09 V, attributing the elevated potential to the weakened Cu–S(Met148) interaction and to the local electrostatic environment of the copper site (Dehabadi et al. 2025). For c-type cytochromes, Multi-Conformation Continuum Electrostatics (MCCE) calculations validated on datasets of up to 141 heme groups spanning 800 mV of redox potential range showed that heme midpoint potentials are collectively modulated by solvent exposure, backbone dipoles, propionate protonation states, and nearby charged residues (Mao et al. 2003, Zheng and Gunner 2009). A complementary MD-based approach combining simulations with Crooks–Bayes fluctuation relations has been applied to heme b-containing proteins, achieving a correlation of 0.85 between calculated and experimental potentials across a series of mutants (Oliveira et al. 2024).

More recently, machine learning methods have emerged as a high-throughput alternative to physics-based calculations, particularly suited to scanning large numbers of cluster variants or protein families. Regression models using structural descriptors such as total cluster charge and average metal valence achieved high accuracy in predicting redox properties across diverse metalloproteins, capturing trends that correlate well with experimental measurements (Min et al. 2025). Dedicated frameworks such as FeS-RedPred extend this approach to enable scalable prediction of reduction potentials specifically for mono- and binuclear iron–sulfur clusters (Persico et al. 2025). While these models currently lack the mechanistic interpretability of quantum-chemical benchmarks and depend critically on the quality and diversity of training data, they represent a promising avenue for rapidly prioritizing variants or identifying functionally important redox tuning in large Fe–S protein families, including those of iron-oxidizing organisms for which experimental measurements remain scarce.

## Applications

Studying the proteins involved in iron oxidation is particularly relevant for elucidating molecular details and optimizing biotechnological processes involving iron-oxidizing microorganisms. Different species of IOB may perform well in removing heavy metals under varying environmental conditions depending on their characteristics and genes. Thus, optimal environmental conditions for different IOB species must be determined to optimize removal performance (Lee et al. 2022). In the following section, the main biotechnological applications involving iron-oxidizing microorganisms or the proteins they produce will be presented.

### Bioleaching

Heap bioleaching is currently the most widely used method in the mining industry for recovering valuable metals such as Cu, Ni, Co, Zn, U, and Au from complex refractory minerals and low-grade ores, due to its energy efficiency and profitability when processing larger volumes of minerals with lower capital costs. Heap bioleaching using acidophilic bacterial consortia is currently being successfully implemented on an industrial scale (López-Martínez et al. 2024).

Conversely, the utilization of microorganisms for extracting metals from electronic waste is a well-studied topic. This approach is considered promising and environmentally friendly for recovering valuable metals from urban solid waste, such as printed circuit boards (PCBs) and lithium-ion batteries (Becci et al. 2020, 2025, Garcia et al. 2025).

For example, in our laboratory, a multi-phase bioleaching system has been developed: starting from the cultivation of *A. ferrooxidans* to produce Fe³⁺ from Fe²⁺, PCBs are then introduced into the culture to initiate metal solubilization, followed by partial medium replacement to reduce metal toxicity and complete the extraction of Cu and Zn. This process achieved 94% and 70% extraction yields for Cu and Zn, respectively, with a further decrease in Fe demand higher than 60% compared to chemical treatment (Becci et al. 2020).

### Wastewater treatment

In wastewater treatment, IOB can catalyze the oxidation of ferrous iron (Fe²⁺) to ferric iron (Fe³⁺), promoting its removal through precipitation and filtration. These microorganisms not only contribute to iron removal but also to the simultaneous elimination of co-occurring contaminants such as manganese, arsenic, and, under specific conditions, nitrogen compounds (Lee et al. 2022). Aziz and colleagues reported enhanced removal efficiency of iron and manganese from groundwater when limestone filters were inoculated with IOB. The presence of these microorganisms also improved the elimination of suspended solids, leading to a significant reduction in turbidity after treatment. When IOB were immobilized on the limestone medium, ferrous iron oxidation and adsorption processes occurred concurrently, facilitating the effective removal of both iron and manganese from the water (Aziz et al. 2020). Indeed, IOB-mediated processes are efficient due to synergistic mechanisms, including biological oxidation, adsorption onto filter media and biofilm formation, which enhances microbial retention and activity (Lee et al. 2022).

### Bioremediation

A different approach is bioremediation, which harnesses microorganisms or their enzymes to capture, convert, or break down environmental contaminants (López-Martínez et al. 2024). A practical application is seen in the treatment of sewage sludge from wastewater treatment plants, using iron- and sulfur-oxidizing microorganisms. Using *A. thiooxidans*, the removal efficiencies were found to be 43.6% for Cr, 96.2% for Cu, 41.6% for Pb, and 96.5% for Zn (López-Martínez et al. 2024).

Another example is the use of a native mixed culture of *A. thiooxidans, A. ferrooxidans, and L. ferrooxidans* to clean up uranium-contaminated soil in Bulgaria. Over 170 days, these acidophiles precipitated the uranium, leaving clean soil in which different plants such as alfalfa, clover, red fescue, and peas could grow (Nicolova et al. 2017).

### Biocatalysis

Bioelectrocatalysis is an interdisciplinary field that combines the selectivity and mild operating conditions of biocatalysis with the energy conversion capabilities of electrochemistry, coupling oxidoreductase enzymes directly to electrode surfaces (Chen et al. 2020). Within this framework, oxidoreductases able to undergo direct electron transfer (DET) with an electrode, without the need for exogenous mediators, represent the most desirable class of biocatalysts, as they enable simpler device architectures, lower operating potentials, and reduced susceptibility to interfering species (Milton and Minteer 2017). DET is considered achievable when the enzyme’s redox cofactor remains bound during catalysis and is located within ~2 nm of the electrode surface (Milton and Minteer 2017). Iron-oxidizing proteins are structurally predisposed to meet these criteria. Outer membrane proteins such as Cyc2 and Cyc2PV-1 present their heme cofactor at the periplasmic face of a transmembrane β-barrel, a geometry that is architecturally compatible with direct interfacial electron transfer. Similarly, the soluble blue copper protein rusticyanin and the HiPIP Iro are compact, single-domain proteins with their redox centers near the protein surface, facilitating electrochemical communication.

These structural features open several lines of biotechnological application. First, iron-oxidizing proteins represent natural candidates for the anodic component of enzymatic fuel cells (EFCs), where Fe²⁺ could serve as the fuel substrate. EFCs exploit the bioelectrocatalytic oxidation of abundant substrates to generate electrical current, and the development of stable bioanodes based on selective iron-oxidizing enzymes would enable energy harvesting in Fe²⁺-rich environments such as acid mine drainage or industrial wastewater streams (Chen et al. 2020). Second, the high substrate specificity of these proteins for soluble Fe²⁺ makes them attractive recognition elements for amperometric biosensors. DET-type enzyme biosensors, as reviewed by Sowa et al. (2026), Keffer et al. (2021), and Jafarpour et al. (2021), eliminate the need for synthetic mediators and allow detection at lower applied potentials, reducing signal interference. A biosensor incorporating an immobilized iron-oxidizing protein could enable real-time, *in situ* monitoring of dissolved Fe²⁺ in environmental or industrial contexts. Third, the intrinsic stability of acidophilic iron-oxidizing proteins under low pH conditions, a direct consequence of their evolutionary adaptation to pH 1–2 environments, constitutes a significant advantage over conventional oxidoreductases, which typically denature under such conditions (Sowa et al. 2026). This stability makes them particularly appealing for bioelectrocatalytic applications in acidic industrial processes.

Despite this potential, several challenges remain to be addressed before practical implementation can be achieved. The primary technical bottleneck is the controlled immobilization of iron-oxidizing proteins onto electrode surfaces in a defined orientation that maximizes DET efficiency, issue that is known to be crucial in biosensing (Rexha et al. 2023, Romagnoli et al. 2023). As highlighted by Milton and Minteer (2017), the dispersion of enzyme orientations at the electrode surface is a major determinant of heterogeneous electron transfer rates, and non-optimal orientations result in significantly reduced catalytic currents. For membrane-associated proteins such as Cyc2, immobilization strategies that preserve the native β-barrel architecture—such as supported lipid bilayers or detergent-stabilized films—will need to be developed and validated. Furthermore, the limited availability of recombinantly produced iron-oxidizing proteins in sufficient quantities for electrode fabrication represents an additional constraint, which recent heterologous expression strategies are beginning to address (Keffer et al. 2021).

## Conclusions and future perspectives

This review has systematically examined the current molecular understanding of proteins directly involved in microbial ferrous iron oxidation, integrating structural, biochemical, and computational perspectives across phylogenetically diverse organisms spanning acidophilic to neutrophilic environments and aerobic to anaerobic metabolic strategies.

The biochemical and structural characterization of iron-oxidizing proteins has advanced significantly over the past two decades, revealing a conserved architectural principle: the spatial segregation of Fe(II) oxidation at or near the cell surface, mediated by specialized redox-active proteins that prevent intracellular iron accumulation and minimize oxidative damage. Despite low sequence identity, monoheme cytochromes such as Cyc2, Cyc572, and Cyc2PV-1 share a common β-barrel porin fold fused to a periplasmic cytochrome domain, while decaheme cytochromes PioA and MtoA adopt extended heme wire architectures anchored by outer membrane β-barrel partners. Copper-containing proteins, including rusticyanin and sulfocyanin, and iron–sulfur proteins such as Iro exemplify alternative cofactor strategies that achieve analogous redox functions under extreme pH conditions.

Computational approaches have emerged as indispensable tools for addressing the experimental challenges inherent to membrane-associated and acidophilic proteins. AlphaFold2 and Rosetta-based homology modeling have provided structural frameworks that guide mutagenesis studies and functional interpretation, while classical molecular dynamics simulations have elucidated protein stability, conformational dynamics and protein–protein interactions within multicomponent electron transfer complexes. Quantum mechanical and hybrid QM/MM methods have enabled the calculation of redox potentials with accuracies approaching 0.1 V, revealing the molecular determinants of cofactor tuning and validating experimentally inaccessible mutants. However, the integration of these computational methods into predictive models of complete electron transfer pathways, from Fe(II) binding at the outer membrane to terminal oxidase activity, remains an open challenge and a priority for future work.

Despite these advances, substantial knowledge gaps persist. Structural information remains limited to computational models for the majority of iron-oxidizing proteins, with few experimentally determined crystal or cryo-EM structures available. The molecular mechanisms underlying the exceptional stability of acidophilic proteins, their resistance to Fe(III)-mediated oxidative stress, and the regulation of iron oxidation pathways in response to environmental fluctuations are incompletely understood. The alternative Iro-mediated pathway in *Acidithiobacillus* spp., although biochemically validated *in vitro*, lacks *in vivo* functional confirmation and a complete electron transfer chain. Moreover, the role of putative accessory proteins and regulatory elements identified through genomic and proteomic analyses awaits experimental characterization.

From a biotechnological perspective, iron-oxidizing proteins present considerable but underexploited potential. Their application in bioleaching, wastewater treatment, and bioremediation has been primarily organism-based, relying on whole-cell processes rather than on the direct utilization of purified enzymes. The recent development of heterologous expression systems for Cyc2PV-1 and Iro represents a critical enabling step toward enzyme-based technologies. In particular, the structural compatibility of iron-oxidizing proteins with DET positions them as promising candidates for bioelectrocatalytic applications, including enzymatic fuel cells and amperometric biosensors for Fe(II) detection. However, realizing this potential requires advances in controlled enzyme immobilization, orientation-dependent electron transfer optimization and long-term operational stability under industrially relevant conditions.

Future research directions should prioritize the experimental determination of high-resolution three-dimensional structures through X-ray crystallography or cryo-electron microscopy, enabling structure-guided engineering of redox properties, substrate specificity and stability. The application of advanced spectroscopic techniques, including time-resolved and single-molecule approaches, will provide mechanistic insights into electron transfer kinetics and conformational dynamics. Expanding computational studies to encompass full electron transfer chains, including explicit treatment of membrane environments and pH gradients, will bridge the gap between molecular structure and physiological function. Finally, the development of scalable recombinant production platforms and rational protein engineering strategies will be essential to translate fundamental knowledge into practical biotechnological applications.

In conclusion, iron-oxidizing proteins represent a functionally diverse and structurally elegant class of redox enzymes that have enabled microbial life in some of Earth’s most extreme environments. Continued interdisciplinary research integrating experimental biochemistry, structural biology, computational modeling and bioengineering will be required to fully unlock their molecular mechanisms and harness their biotechnological potential for sustainable metal recovery, environmental remediation, and bioelectrochemical energy conversion.

## Supplementary Material

fuag055_Supplemental_File

## Data Availability

The data underlying this article are available in the article and in its online supplementary materials
