# Supplementary material for: Biomolecular mechanisms of iron oxidation in prokaryotes: a protein focused review
Source: FEMS Microbiol Rev. 2026 Nov 3;50:fuag055. doi: 10.1093/femsre/fuag055 (PMC13741234; doi:10.1093/femsre/fuag055)
Supplement: Supplementary file 1 [file fuag055_supplemental_file.pdf]

Biomolecular Mechanisms of Iron Oxidation in prokaryotes: a protein focused review

Gianmarco D'Antonio[1#], Agnese Roscioni[1#], Alice Romagnoli[1,2], Alessandro Becci[1], Francesca Beolchini[1], Giovanni Di Muccio[1,2]*, Alessia Amato[1]*, Anna La Teana[1,2]

1. Department of Life and Environmental Sciences, Polytechnic University of Marche, Via Brecce Bianche, 60131, Ancona, Italy
2. New York-Marche Structural Biology Center (NY-MaSBiC), Department of Life and Environmental Sciences, Polytechnic University of Marche, Via Brecce Bianche, 60131 Ancona, Italy

#These authors contributed equally to the work

* Corresponding Author: Alessia Amato email: a.amato@staff.univpm.it, g.dimuccio@staff.univpm.it

Supplementary Information

Table of content

Glossary	3
**Table S1** Overview of the proteins involved in iron oxidation pathways discussed in this review	4
Material and Methods, computational modelling	5
**Figure S1**. AlphaFold 3 models of the monoheme cytochromes c	7
**Table S2**. Confidence of the AlphaFold 3 models of Cyc2, Cyt572 and Cyc2PV-1	7
**Figure S2**. SWISS-MODEL homology model of sulfocyanin	8
**Table S3**. SWISS-MODEL homology model of sulfocyanin (template rusticyanin, 1RCY)	8
**Figure S3**. AlphaFold 3 models of FoxC and of the FoxC–FoxD complex	9
**Table S4**. Confidence of the AlphaFold 3 models of FoxC and of the FoxC–FoxD complex	9
**Figure S4**. AlphaFold 3 models of the decaheme cytochrome–porin complexes.	10
**Table S5**. Confidence of the AlphaFold 3 models of the PioA–PioB and MtoA–MtoB complexes	10
**Figure S5**. SWISS-MODEL homology model of Iro.	11
**Table S6**. SWISS-MODEL homology model of Iro (template HiPIP, 1ISU)	11

Glossary

Aerobic: Requiring or using molecular oxygen for growth and energy metabolism.

Acidophilic: Exhibiting a maximum growth rate in highly acidic environments, with an optimal pH equal to or lower than 2.

Chemoautotroph/Chemolithoautotroph/Lithotroph: Microorganism that derives the energy and reducing power necessary for survival from the oxidation of inorganic chemical compounds, using carbon dioxide as the main carbon source for the synthesis of cellular organic molecules.

Thermophily: Physiological condition and ecological adaptation allowing certain organisms (thermophiles) to develop and reproduce at high temperatures, typically between 45 °C and 80 °C, with an optimal growth temperature around 55 °C.

Mesothermophilic: Operating at moderately high temperatures, typically between 40 °C and 55 °C; often referring to formally mesophilic bacteria possessing a marked tolerance or preference for the medium-high temperatures of this range.

Thermoacidophilic: Combining adaptation to high temperatures (thermophily) with adaptation to highly acidic environments (acidophily).

Neutrophilic: Exhibiting a maximum growth rate in environments with a neutral or near-neutral pH, typically between 5.5 and 8.0, with an ideal optimum around pH 7.0.

Anaerobic: Not requiring molecular oxygen for energy metabolism and growth.

Photoautotrophic: Deriving energy from sunlight and using carbon dioxide (CO2, inorganic carbon) as the main carbon source for the synthesis of organic molecules.

Photoheterotrophic: Deriving energy from sunlight through photosynthesis and using pre-existing organic compounds, rather than carbon dioxide, as the main carbon source for the synthesis of cellular constituents.

Chemoheterotrophic: Using organic chemical compounds both as an energy source and as the main carbon source.

Table S2. Overview of the proteins involved in iron oxidation pathways discussed in this review.

Organism	Protein / Complex	Functional Category	Evidence for iron oxidation
Acidithiobacillus spp.	**Cyc2**	Iron-oxidizing protein	Experimentally validated: purified protein; Fe(II)-dependent reduction; spectroscopic characterization; mutagenesis (Yarzábal A. et al. 2002, Castelle C et al. 2008, Su et al. 2025)
	Rusticyanin (Rus)	Electron transfer protein	
	Cyc1 / CycA1	Electron transfer protein	
	Cup	Electron transfer protein	
	CoxABCD	Metabolic pathway-associated proteins	
	**Iro (HiPIP)**	Iron-oxidizing protein / Candidate	Experimentally validated in *A. Ferridurans* strain: heterologous expression; Fe(II)-dependent electron transfer; kinetic analysis (Liu Y et al. 2023)
	Hip	Metabolic pathway-associated protein	
	Bc$_1$ complex (petI/petII)	Metabolic pathway-associated proteins	
	NADH dehydrogenase I	Metabolic pathway-associated protein	
Leptospirillum spp.	**Cyt572**	Iron-oxidizing protein	Experimentally validated: purified protein; spectroscopic and redox characterization (Jeans et al. 2008)
	Cyt579	Electron transfer protein	
	Soluble c-type cytochrome	Electron transfer protein	
	Cytochrome c oxidase (cbb_3)	Metabolic pathway-associated protein	
	bc$_1$ complex & NADH DH	Metabolic pathway-associated proteins	
Metallosphaera spp.	**FoxC**	Iron-oxidizing protein	Inferred from indirect evidence: proteomic identification; Fe(II)-dependent transcriptional upregulation; comparative genomics; structural homology (Counts, Vitko, and Kelly 2022, Kozubal et al. 2011)
	FoxD	Electron transfer protein / Structural partner	
	Multicopper oxidase (Mco)	Electron transfer protein	
	FoxAB	Metabolic pathway-associated proteins	
	bc complex & NADH DH	Metabolic pathway-associated proteins	
Ferroplasma spp.	**Sulfocyanin**	Iron-oxidizing protein	Experimentally supported at the complex level: Fe(II)-oxidizing activity and O_2 consumption in sulfocyanin-containing complexes. (Castelle CJ et al. 2015)
	Heme/copper oxidase	Metabolic pathway-associated protein	
	Rieske cytochrome b complex	Electron transfer / Metabolic pathway protein	
Mariprofundus ferrooxydans	**Cyc2PV-1**	Iron-oxidizing protein	Experimentally validated: proteomic identification; heterologous expression; Fe(II)-dependent reduction; spectroscopic characterization (Barco et al. 2015, Keffer et al. 2021)
	Cyc1PV-1	Electron transfer protein	
	Mob (ActB)	Candidate inferred from expression/homology	
	Terminal oxidases	Metabolic pathway-associated protein	
	bc$_1$ complex / ACIII	Metabolic pathway-associated proteins	
	NADH dehydrogenase	Metabolic pathway-associated protein	
Sideroxydans lithotrophicus	**MtoA**	Iron-oxidizing protein	Experimentally validated: heterologous expression; Fe(II)-dependent reduction; ligand-dependent Fe(II) oxidation activity (Liu J et al. 2012)
	MtoB	Structural partner	
	MtoD	Electron transfer protein	
	CymA	Electron transfer protein	
	Cyc2 (3 isoforms)	Iron-oxidizing protein / Candidate	
Rhodopseudomonas palustris TIE-1	**PioA**	Iron-oxidizing protein	Experimentally validated: gene deletion; loss of Fe(II)-oxidizing activity; biochemical characterization; Fe(II)-dependent reduction (Jiao and Newman 2007, Gupta et al. 2019)
	PioB	Structural partner	
	PioC	Electron transfer protein	
	Reaction center (LH-RC)	Metabolic pathway-associated proteins	
	bc$_1$ complex	Metabolic pathway-associated proteins	

Material and Methods, computational modelling

S1. Structure prediction and homology modelling

Sequences. Protein sequences were retrieved from the UniProt and NCBI databases. Identifiers are given as organism (strain, where relevant); locus tag; database accession. Cyc2 (*Acidithiobacillus ferrooxidans*; NCBI ACK79618.1); Cyt572 (*Leptospirillum* sp.; NCBI WP_231587394.1); Cyc2PV-1 (*Mariprofundus ferrooxydans* PV-1; NCBI AKN35166.1, UniProt A0A0H3ZGZ2); FoxC (*Metallosphaera sedula*; Msed_0478; UniProt A4YE03); FoxD (*M. sedula*; Msed_0477; UniProt A4YE02); sulfocyanin (*Ferroplasma sp.*; UniProt A0A1V0N1W7); PioA (*Rhodopseudomonas palustris* TIE-1; UniProt A1EBT2); PioB (*R. palustris* TIE-1; UniProt A1EBT3); MtoA (*Sideroxydans lithotrophicus* ES-1; Slit_2497; UniProt D5CMQ0); MtoB (*S. lithotrophicus* ES-1; Slit_2496; UniProt D5CMP9); Iro (*A. ferrooxidans*; UniProt P50500). Residue numbering follows the UniProt entries throughout (signal peptides included).

Protocol. Template availability was first assessed with SWISS-MODEL (Section S1.2). Proteins for which a template with adequate coverage was available (sulfocyanin and Iro) were modeled by homology with SWISS-MODEL. All other targets were predicted with AlphaFold 3, either because suitable templates were unavailable or did not encompass the cofactor-binding regions (Cyc2, Cyt572, Cyc2PV-1, FoxC) or because multi-subunit assemblies with cofactors had to be modeled (FoxC–FoxD, PioA–PioB, MtoA–MtoB). Template searches are summarized in Tables S2 and S5 and in Section S1.2; homology-model details in Tables S3 and S6.

S1.1 AlphaFold 3.

Predictions were run on the AlphaFold Server (AlphaFold 3; Abramson et al., 2024) with default settings: one random seed per job and five diffusion samples per seed. Heme cofactors were included in each job as ligands. For the c-type cytochromes, heme C (CCD code HEC) was added: one HEC for Cyc2, Cyt572 and Cyc2PV-1, and ten HEC bound to the A chain (PioA, MtoA) in the PioA–PioB and MtoA–MtoB complexes. For the b-type cytochrome FoxC, one heme B (CCD code HEM) was added, both for the isolated protein and for the FoxC–FoxD complex. For each job, the highest-ranking model was used in all figures; the ranking score combines pTM and ipTM with penalties for disordered residues and clashes (Abramson et al., 2024). The five models of each job were mutually consistent (ranking scores are reported in Tables S2, S4 and S5).

Model confidence was assessed with three metrics: (i) the per-residue pLDDT, reported both as the mean over each chain and as the mean over the residues within 4 Å of each cofactor; (ii) pTM and ipTM—for single-chain jobs containing a ligand, ipTM reflects the protein–ligand interface rather than a protein–protein one; (iii) the predicted aligned error (PAE), reported for the complexes as the mean inter-chain PAE. In Figs S1, S3 and S4, pLDDT is color-coded with the standard AlphaFold scale. Heme iron coordination was evaluated in the top-ranked model from the Fe–N/S distances to candidate axial ligands (cut-off 2.8 Å), excluding backbone nitrogen atoms.

S1.2 SWISS-MODEL template search and homology modelling

Template identification and, where applicable, model building were performed with SWISS-MODEL (Waterhouse et al., 2018, Studer et al., 2021) on 5 October 2026, using the target sequences listed in Section S1. For each protein, the best-ranking template according to SWISS-MODEL was considered; template, sequence coverage and sequence identity are reported in Tables S2 and S5. For the two homology models used in the figures (sulfocyanin and Iro), GMQE and QMEAN4 z-score are also reported (Tables S3 and S6). Local model quality was assessed with QMEANDisCo (Studer

et al., 2020); the per-residue scores, stored in the B-factor column of the SWISS-MODEL output files, were used for coloring (Figs S2 and S5).

For Cyc2, Cyt572 and Cyc2PV-1, the best-ranking templates (porin O, PDB 4RJX, for Cyc2 and Cyt572; cis-aconitate porin OpdH, PDB 3T20, for Cyc2PV-1) shared 8.8–14.4% sequence identity with the targets and did not cover the N-terminal cytochrome domain that carries the heme-binding motif: the modeled regions were residues 92–485, 136–516 and 248–416, respectively, whereas the CXXCH motifs span residues 43–47, 40–44 and 43–47. For FoxC and FoxD, the best templates (PDB 2IVF and 7DUW) covered only 10.2% and 26.7% of the sequence, respectively. PioA, PioB, MtoA and MtoB could be modeled individually by homology (coverage 48–83%), but SWISS-MODEL models neither the hemes nor the PioA–PioB and MtoA–MtoB interfaces. In all these cases, the AlphaFold 3 prediction was used.

S1.3 Cofactor placement in the homology models

In the sulfocyanin and Iro models, the cofactors were transferred from the respective templates by SWISS-MODEL: the copper ion from rusticyanin (PDB 1RCY) and the [Fe4S4] cluster of the predicted Iro protein, from the HiPIP of *Rhodocyclus tenuis* (PDB 1ISU). Cofactor positions in these two models are therefore inherited from the template rather than predicted, and no refinement was performed. For sulfocyanin, the copper-coordinating residues (His82, Cys145, His150, Met155) were identified from the Cu–N/S distances (cut-off 2.8 Å, as in Section S1.1).

S1.4 Hemes in the PioA–PioB and MtoA–MtoB complexes

PioA–PioB and MtoA–MtoB were predicted as protein–protein complexes in jobs that include ten heme C ligands on the A chain (Table S5); heme positions and the His/His and His/Met coordination reported in the main text (Fig. 6) are taken from these predictions.

S1.5 Visualisation

Structures were visualised and rendered with UCSF ChimeraX 1.10.1 (Meng *et al*., 2023). AlphaFold 3 models were coloured by pLDDT (colour scales next to each figure); SWISS-MODEL models by local QMEANDisCo. Heme and Cu cofactors are shown as sticks and spheres, respectively. PAE plots were generated from the files of the server output.

References

- Abramson J, Adler J, Dunger J *et al.* Accurate structure prediction of biomolecular interactions with AlphaFold 3. *Nature* 2024;**630**(8016):493–500. https://doi.org/10.1038/s41586-024-07487-w.
- Meng EC, Goddard TD, Pettersen EF et al. UCSF ChimeraX: Tools for structure building and analysis. Protein Sci 2023;32(11):e4792. https://doi.org/10.1002/pro.4792.
- Studer G, Rempfer C, Waterhouse AM et al. QMEANDisCo—distance constraints applied on model quality estimation. Bioinformatics 2020;36(6):1765–71. https://doi.org/10.1093/bioinformatics/btz828.
- Studer G, Tauriello G, Bienert S et al. ProMod3—A versatile homology modelling toolbox. PLoS Comput Biol 2021;17(1):e1008667. https://doi.org/10.1371/journal.pcbi.1008667.
- Waterhouse A, Bertoni M, Bienert S *et al.* SWISS-MODEL: Homology modelling of protein structures and complexes. *Nucleic Acids Res* 2018;**46**(W1):W296–303. https://doi.org/10.1093/nar/gky427.

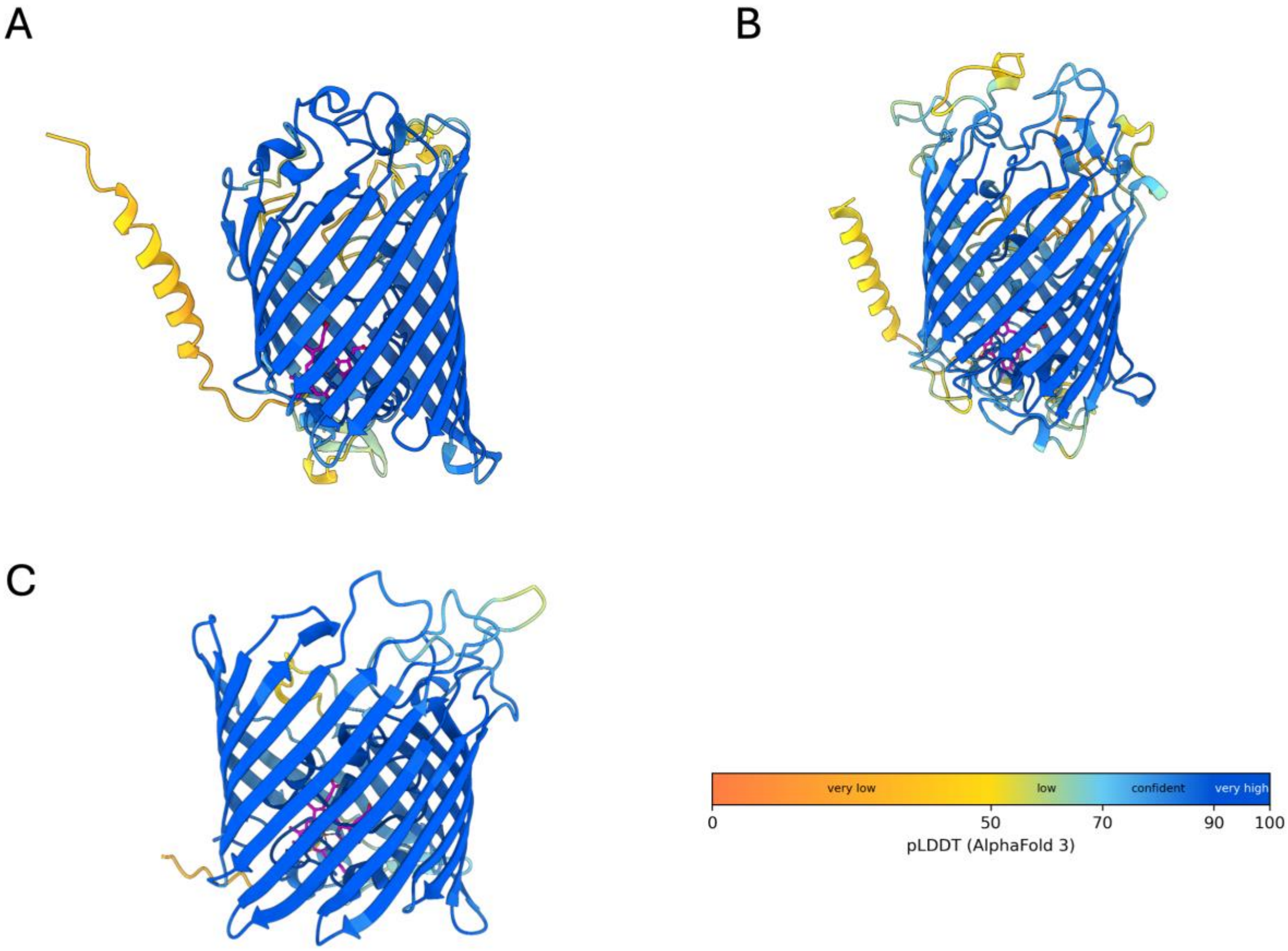

Figure S6. **AlphaFold 3 models of the monoheme cytochromes c. A**. Cyc2 (*Acidithiobacillus ferrooxidans*), **B.** Cyt572 (*Leptospirillum* sp.) and **C.** Cyc2PV-1 (*Mariprofundus ferrooxydans).* Models are coloured by per-residue confidence (pLDDT; scale at the bottom: orange <50, yellow 50–70, light blue 70–90, dark blue >90). The heme C cofactor (HEC, one per model) is shown as magenta sticks. Structures are oriented with the β-barrel axis vertical and the cytochrome domain at the bottom.

Table S2. Confidence of the AlphaFold 3 models of Cyc2, Cyt572 and Cyc2PV-1

**Protein**	**Ranking score**	**pTM / ipTM**	**Mean pLDDT**	**Heme-site pLDDT**	**Fe axial ligand**	**SWISS-MODEL best template; coverage / identity**
**Cyc2**	0.96	0.85 / 0.96	79.9	80.6	His47	4RJX; 68% / 12%
**Cyt572**	0.94	0.82 / 0.95	74.0	76.8	His44	4RJX; 51% / 9%
**Cyc2PV-1**	0.95	0.85 / 0.95	81.2	77.8	His47	3T20; 35% / 14%

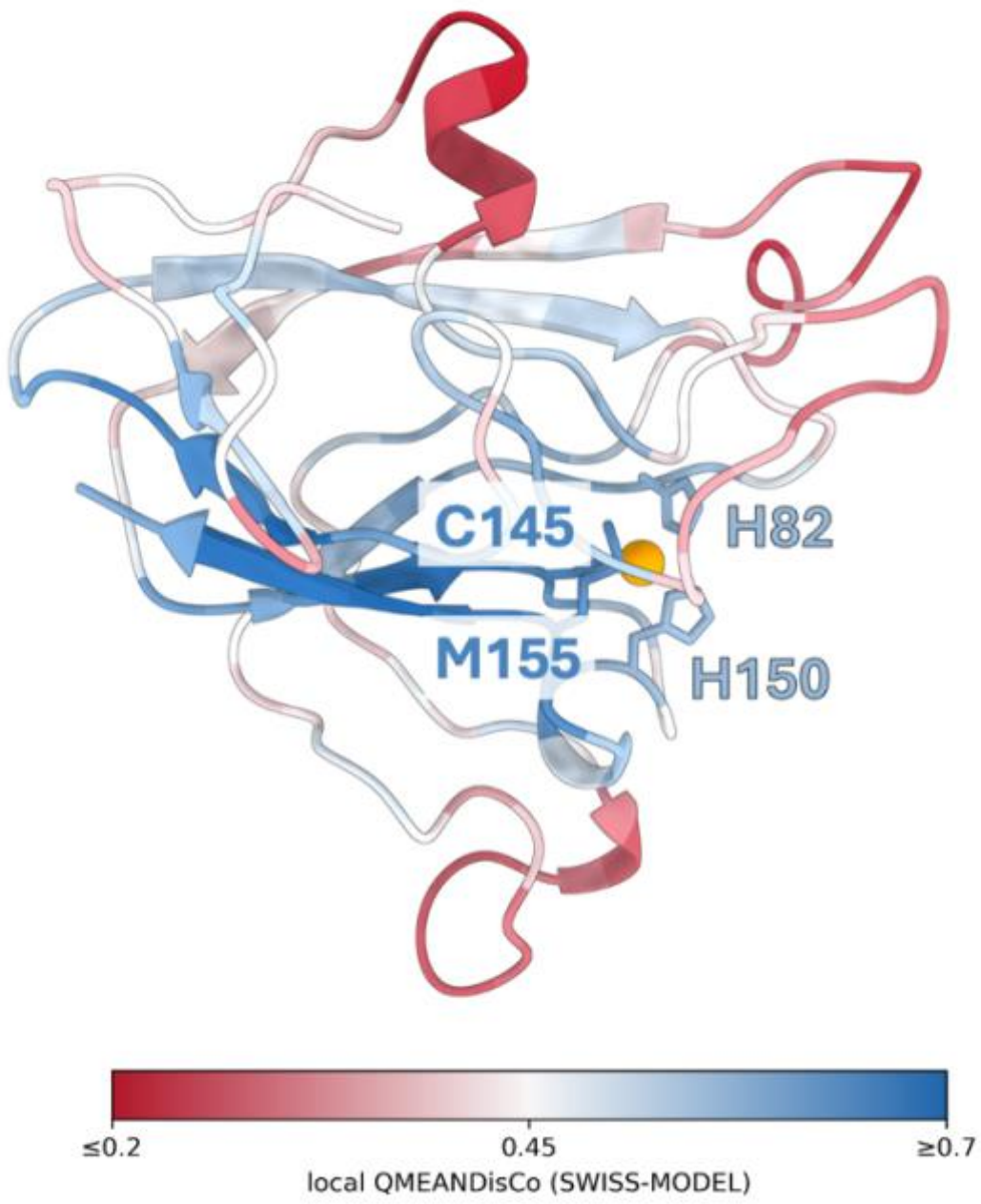

Figure S7. **SWISS-MODEL homology model of sulfocyanin**. Model of sulfocyanin (*Ferroplasma sp.)* built on rusticyanin (PDB 1RCY). The structure is coloured by local model quality (QMEANDisCo; scale at the bottom: red ≤0.2, white 0.45, blue ≥0.7). The Cu ion is represented as orange sphere, and its coordinating residues (His82, Cys145, His150 and Met155 UniProt numbering) are shown as sticks.

Table S3. SWISS-MODEL homology model of sulfocyanin (template rusticyanin, 1RCY)

Coverage (%)	Identity (%)	GMQE	QMEAN4 z	Cu ligands (UniProt numbering)	Local QMEANDisCo at site
**75.1**	22.3	0.41	-4.83	His82, Cys145, His150 and Met155	0.57–0.66 (model mean 0.45)

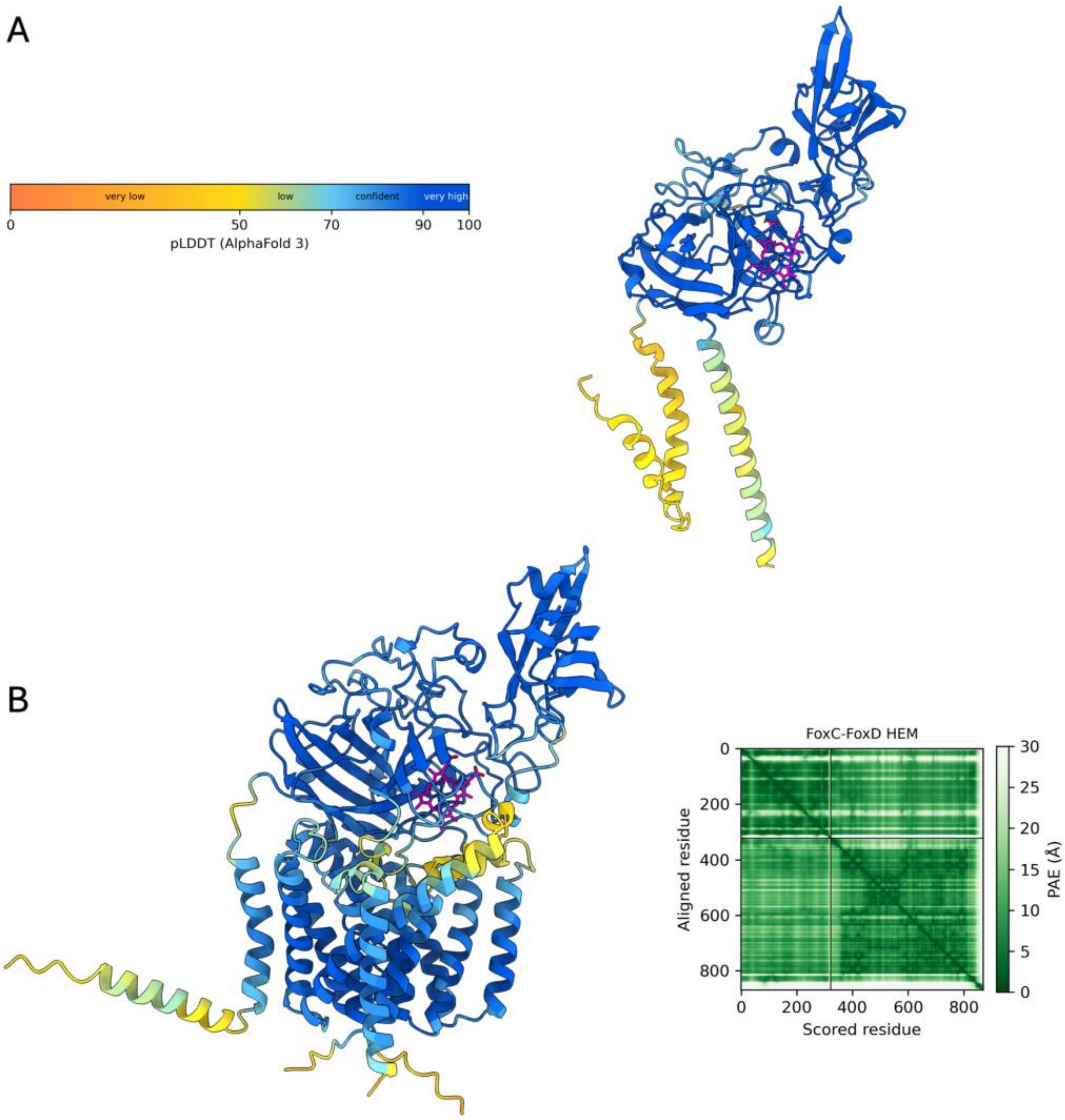

Figure S8. **AlphaFold 3 models of FoxC and of the FoxC–FoxD complex. A.** FoxC (*Metallosphaera sedula*) predicted with one heme B (HEM); the heme is depicted with magenta sticks. **B.** FoxC–FoxD complex predicted with one heme B bound to FoxC; right, predicted aligned error (PAE) of the complex (FoxD, residues 1–322; FoxC, residues 323–870; black lines separate the chains; scale 0–30 Å). Structures are coloured by pLDDT (scale at the top). Complex: ipTM 0.77, FoxD–FoxC interface ipTM 0.75, mean interface PAE 12.7 Å. The Lys478–Fe distance in the complex (1.46 Å) is shorter than expected for a Fe–N bond and the Lys478 ligation should be considered tentative.

Table S4. Confidence of the AlphaFold 3 models of FoxC and of the FoxC–FoxD complex.

**Model**	**Ranking score**	**pTM**	**Interface ipTM (FoxD–FoxC)**	**Interface PAE (Å)**	**Mean pLDDT (FoxD / FoxC)**	**Heme-site pLDDT**	**Fe axial ligands**	**SWISS-MODEL best template; coverage / identity**
**FoxC**	0.98	0.82	–	–	84.3	93.9	Lys478; Met296	FoxC: 2IVF; 10% / 30%
**FoxC–FoxD**	0.82	0.82	0.75	12.7	78.2 / 82.3	81.8	Lys478; Met296	FoxD: 7DUW; 27% / 16%<br>FoxC: 2IVF; 10% / 30%

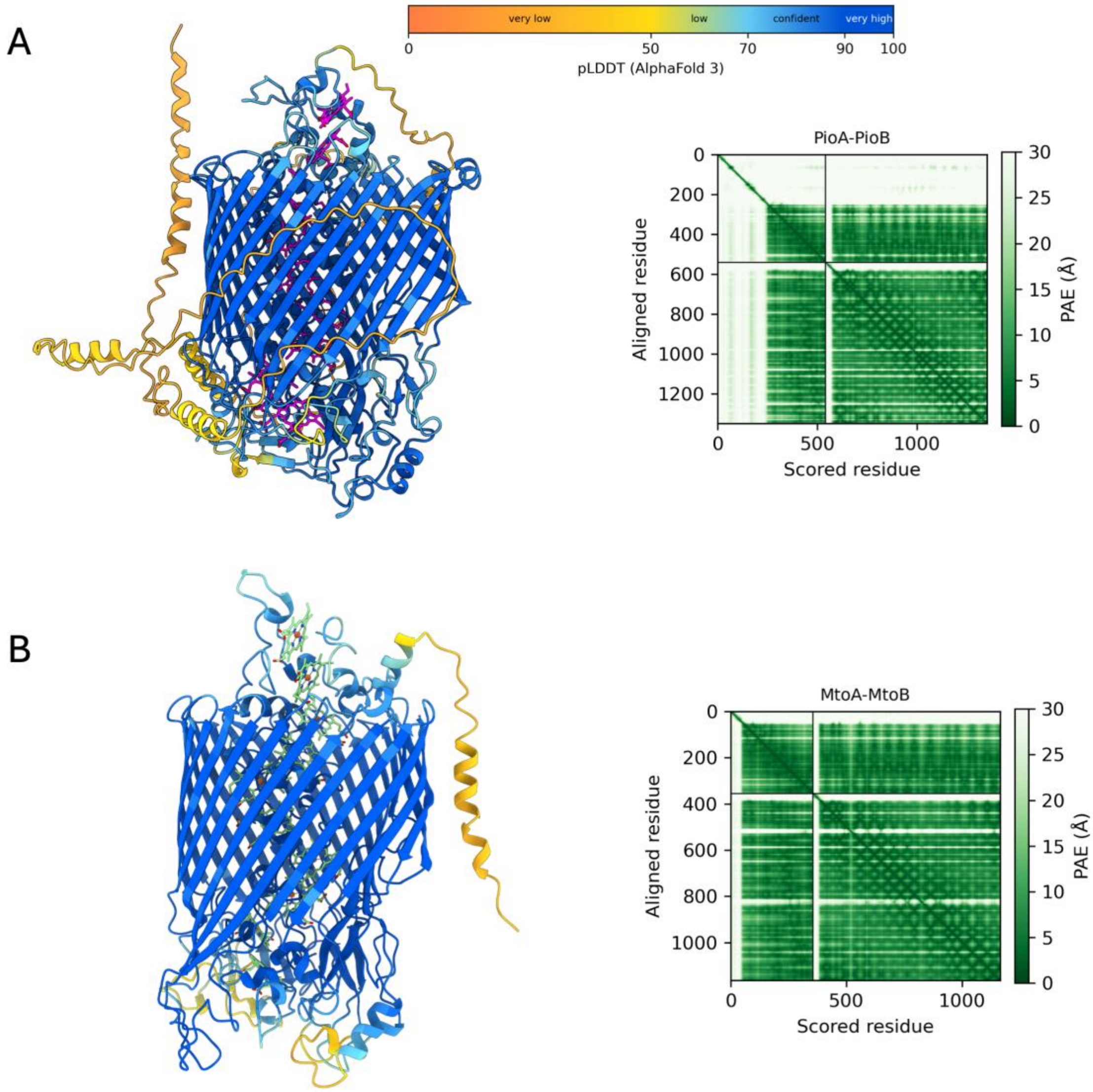

Figure S9. **AlphaFold 3 models of the decaheme cytochrome–porin complexes. A.** PioA–PioB (*Rhodopseudomonas palustris*) and **B.** MtoA–MtoB (*Sideroxydans lithotrophicus),* each predicted as a complex with ten heme C (HEC) bound to chain A (magenta sticks). Left, structures coloured by pLDDT (scale at the top); right, PAE of the complex (chain A on the first axis, then chain B; black lines separate the chains; scale 0–30 Å). Interface ipTM and mean interface PAE are 0.86 and 19.1 Å (PioA–PioB) and 0.87 and 12.1 Å (MtoA–MtoB).

Table S5. Confidence of the AlphaFold 3 models of the PioA–PioB and MtoA–MtoB complexes.

Complex	Ranking score	pTM	Interface ipTM (A–B)	Interface PAE (Å)	Mean pLDDT (A / B)	Heme-site pLDDT range	Heme axial ligation	SWISS-MODEL (6R2Q); coverage / identity
**PioA–PioB**	0.98	0.80	0.86	19.1	58.1 / 82.7	77–90	3× His/Met, 7× His/His	A: 48% / 38% B: 80% / 19%
**MtoA–MtoB**	0.94	0.88	0.87	12.1	79.3 / 85.3	85–94	10× His/His	A: 75% / 40% B: 83% / 19%

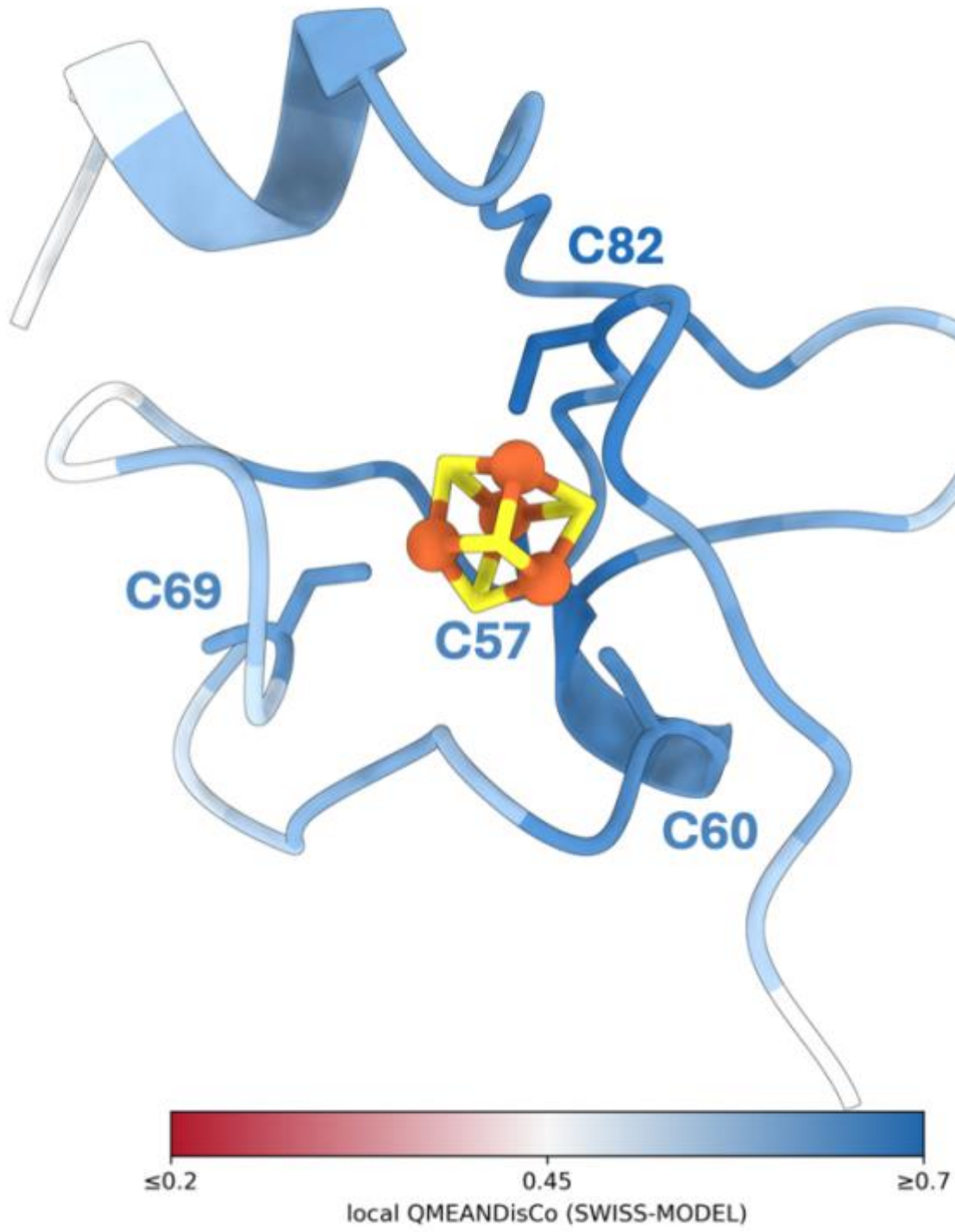

Figure S10. **SWISS-MODEL homology model of Iro**. Model of Iro (*A. ferrooxidans*) built on the high-potential iron–sulfur protein of *Rhodocyclus tenuis* (PDB 1ISU). The structure is coloured by local model quality (QMEANDisCo; scale at the bottom: red ≤0.2, white 0.45, blue ≥0.7). The [Fe_4S_4] cluster and coordinating cysteines (Cys57, Cys60, Cys69 and Cys82, mature Cys20/23/32/45) are represented as sticks.

Table S6. SWISS-MODEL homology model of Iro (template HiPIP, 1ISU)

Coverage (%)	Identity (%)	GMQE	QMEAN4 z	Cys ligands (Uniprot Numbering)	Local QMEANDisCo at Cys
**57.8**	44.2	0.38	-2.81	Cys57, Cys60, Cys69, Cys82 (mature Cys20/23/32/45)	0.63–0.72
